# Histone chaperone ASF1A accelerates chronic myeloid leukemia blast crisis by activating Notch signaling

**DOI:** 10.1038/s41419-022-05234-5

**Published:** 2022-10-03

**Authors:** Xiaolin Yin, Minran Zhou, Lu Zhang, Yue Fu, Man Xu, Xiaoming Wang, Zelong Cui, Zhenxing Gao, Miao Li, Yuting Dong, Huimin Feng, Sai Ma, Chunyan Chen

**Affiliations:** 1 Department of Hematology, Qilu Hospital, Shandong University, Jinan, Shandong China; 2 Department of Physiology & Pathophysiology, School of Basic Medical Science, Cheeloo College of Medicine, Shandong University, Jinan, Shandong China

**Keywords:** Chronic myeloid leukaemia, Cell signalling

## Abstract

The blast crisis (BC) is the final deadly phase of chronic myeloid leukemia (CML), which remains a major challenge in clinical management. However, the underlying molecular mechanism driving blastic transformation remains unclear. Here, we show that ASF1A, an essential activator, enhanced the transformation to CML-BC by mediating cell differentiation arrest. ASF1A expression was aberrantly increased in bone marrow samples from CML-BC patients compared with newly diagnosed CML-chronic phase (CP) patients. ASF1A inhibited cell differentiation and promoted CML development in vivo. Mechanistically, we identified ASF1A as a coactivator of the Notch transcriptional complex that induces H3K56ac modification in the promoter regions of Notch target genes, and subsequently enhanced RBPJ binding to these promoter regions, thereby enhancing Notch signaling activation to mediate differentiation arrest in CML cells. Thus, our work suggests that targeting ASF1A might represent a promising therapeutic approach and a biomarker to detect disease progression in CML patients.

## Introduction

Chronic myeloid leukemia (CML) is a myeloproliferative disorder characterized by the translocation of chromosomes 9 and 22, consequently expressing the fusion oncogene *BCR-ABL*, which results in constitutive tyrosine kinase activity [1–3]. CML proceeds in three phases: chronic phase (CP), accelerated phase (AP), and blast crisis (BC) [4]. CML is usually diagnosed during CP, which increases the proliferation of myeloid cells without losing their capacity to differentiate. However, the disease inevitably progresses to the AP and ultimately to the invariably fatal BC, which is characterized by the rapid expansion of myeloid or lymphoid differentiation-arrested blast cells with high resistance to chemotherapy and short survival [5]. Thus, differentiation blockage in progenitor stages is the mainstay of BC phase progression [5, 6]. However, the exact molecular mechanism of differentiation arrest-induced CML-CP transformation to CML-BC remains largely unknown.

Notch signaling plays a critical role in controlling the development and adult tissue homeostasis under normal physiological conditions [7]. Accumulating evidence demonstrates that Notch signaling is widely and constitutively overexpressed in various malignancies, including CML [8, 9]. Upon Notch signaling activation, the intracellular domain of the Notch protein (NICD) translocates into the nucleus and binds to its transcriptional effector, recombination signal-binding immunoglobulin kappa J region (RBPJ), resulting in the transcription of Notch target genes, such as the proto-oncogene *c-Myc* and hairy enhancer of split 1 (*HES1*), which are associated with the differentiation of hematopoietic cells and CML-BC transformation [8, 10, 11]. Therefore, understanding how Notch signaling is aberrantly activated to drive CML-BC transformation is particularly important.

Histone chaperone complexes are crucial for histone folding, oligomerization, post-translational modification, and chromosomal processes [12]. Dysregulation of histone chaperones plays a key role in leukogenesis and tumorigenesis [13–15]. For example, deletion of the histone chaperone chromatin assembly factor 1 B (*CHAF1B*) gene drives AML cell differentiation and prevents leukemia development [16]. The histone chaperone FACT complex mediates an expeditious oxidative stress response that promotes liver cancer progression [17]. Anti-silencing function 1 (ASF1) is a key histone H3-H4 chaperone that interacts with newly synthesized H3-H4 heterodimers and includes two distinct isoforms, ASF1A and ASF1B. Both isoforms are involved in DNA replication-coupled and DNA replication-independent nucleosome assembly pathways [18, 19]. Interestingly, ASF1A is specifically required for the acetylation of histone H3 on lysine 56 (H3K56ac), but not ASF1B [20, 21]. However, the biological functions of ASF1 in CML transformation remain unknown.

In this study, we show that ASF1A facilitates Notch signaling activation to induce differentiation arrest in CML cells by enhancing H3K56ac. ASF1A is significantly elevated in CML-BC patients compared to CML-CP patients, suggesting that ASF1A may contribute to CML transformation. We identified ASF1A as a coactivator with RBPJ to induce H3K56ac modification in the promoter regions of Notch target genes, thereby enhancing RBPJ binding to these promoter regions. Our work clarifies how ASF1A acts as a molecular switch to control the transformation from CML-CP to CML-BC and uncovers a novel mechanism of Notch signaling activation, thereby targeting ASF1A might represent a promising therapeutic approach and a biomarker to detect phase progression in CML patients.

## Materials and methods

### Cells

K562 and MEG01 cell lines were obtained from and authenticated by the Typical Culture Preservation Commission Cell Bank, Chinese Academy of Sciences (Shanghai, China). These cells were cultured in RPMI 1640 medium supplemented with 10% fetal bovine serum (FBS; Gibco, Carlsbad, CA, USA) without antibiotics.

### Patient characteristics and sample preparation

Bone marrow samples were obtained from patients with newly diagnosed CML-CP (*n* = 43) and CML-BC (*n* = 24). Patients were evaluated at the Department of Hematology, Qilu Hospital of Shandong University, Jinan, China. The informed consent was obtained from all subjects. The clinical characteristics of these patients (*n* = 67) are listed in Supplementary Table 1. Mononuclear cells were isolated from the samples and stored at −80 °C. This study was approved by the Ethics Committee of Qilu Hospital of Shandong University.

### RNA extraction and quantitative reverse-transcription PCR (qRT-PCR)

Total RNA from human bone marrow samples or cultured cells was extracted using TRIzol reagent (Invitrogen, Carlsbad, CA, USA). Isolated RNA was used as a template for first-strand cDNA synthesis, following the manufacturer’s protocol (RevertAid RT kit, Fermentas, Canada). Expressions of *ASF1A*, *c-myc*, and *HES1* mRNAs were quantified by qPCR using the SYBR Premix Ex Taq kit (Takara, Japan). Gene expression was normalized to their respective actin or β2M levels (housekeeping reference genes). Gene expression was calculated using the 2^−ΔΔCT^ method. The sequences of the primers used are listed in Supplementary Table 2.

### Western blotting

Cells were collected, washed twice in phosphate-buffered saline (PBS), and then lysed for 30 min on ice in RIPA buffer supplemented with 1 mM phenylmethylsulfonyl fluoride (PMSF). Total cellular proteins were separated by sodium dodecyl sulfate-polyacrylamide gel electrophoresis (SDS-PAGE) and transferred to polyvinylidene fluoride (PVDF) membranes. Primary antibodies against ASF1A (1:1000, Cell Signaling Technology, Cat#2990), c-Myc (1:1000, Abcam, Cat#ab56), HES1 (1:1000, Cell Signaling Technology, Cat#11988), and actin (1:10000, Sigma) were incubated with the membranes at 4 °C overnight. Horseradish peroxidase-conjugated anti-rabbit and anti-mouse secondary antibodies (Jackson ImmunoResearch, USA) were diluted to 1:4000 and incubated with the respective membranes at room temperature for 50 min. Protein blots were visualized with enhanced chemiluminescence reaction (ECL+, Millipore, USA).

### Co-immunoprecipitation (Co-IP) and immunoblot analyses

Protein extracts were incubated with 5 μg of antibody. Co-IP analysis was performed according to the manufacturer’s protocol (Pierce TM Co-Immunoprecipitation Kit, Thermo Fisher Scientific, Waltham, MA, USA). Primary antibodies against ASF1A (1:1000, Cell Signaling Technology, Cat#2990) and RBPJ (1:1000, Cell Signaling Technology, Cat #5313) were used for immunoblot detection.

### Chromatin immunoprecipitation (ChIP) assay

K562 and MEG01 cells were treated according to the manufacturer’s protocol (ChIP Assay Kit, Cell Signaling Technology, USA). The cells were cross-linked with 37% formaldehyde solution for 10 min at 37 °C, and then sonicated to develop soluble chromatin with DNA fragments (ranging in size from 200 to 800 bp). DNA was purified from chromatin fragments immunoprecipitated with antibodies against ASF1A and RBPJ (Cell Signaling Technology). The purified DNA was used for PCR amplification. The respective PCR primers are listed in Supplementary Table 2.

### Immunohistochemistry (IHC)

Paraffin-embedded slides were deparaffinized and rehydrated, followed by antigen retrieval using citric acid buffer. Endogenous peroxidase was deactivated with hydrogen peroxide. Slides were blocked using 10% goat serum and then incubated with the corresponding primary antibodies overnight at 4 °C. After incubation with secondary antibodies for 30 min at room temperature, 3,3’-diaminobenzidine (DAB) staining (Thermo Fisher Scientific) was used to detect antigen-antibody binding. The primary antibodies used were as follows: ASF1A (1:100, Cell Signaling Technology, Cat#2990), c-Myc (1:50, Abcam, Cat#ab56), and HES1 (1:50, Cell Signaling Technology, Cat#11988).

### Immunostaining

Mononuclear cells isolated from bone marrow samples were used to prepare cell smears with poly-l-lysine (PLL)-treated glass slides and then fixed in ice-cold acetone. Samples were incubated with anti-ASF1A antibody (1:200, Cell Signaling Technology, Cat#2990) overnight at 4 °C, followed by incubation with horseradish peroxidase-conjugated secondary antibody for 30 min.

### Luciferase reporter assays

K562 and MEG01 cells were infected with lentiviruses expressing shRNA for *ASF1A* (sh*ASF1A-1*) or a non-specific scrambled control (sh*NC*), and then screened with puromycin to obtain cells that stably inhibited *ASF1A* expression. Next, these cells were transfected with firefly luciferase vectors containing the wild-type or mutant *c-Myc* and *HES1* promoters. A *Renilla* luciferase reporter plasmid containing the thymidine kinase promoter (TK), was co-transfected to assess transfection efficiency. After 48 h, luciferase activity was measured using the Dual-Luciferase Reporter Assay System (Promega, USA). Firefly luciferase activity was normalized to the respective TK-mediated Renilla activity.

### Lentiviral transduction

Pre-made lentiviruses (purchased from Shanghai GenePharma Co., Ltd, China) were concentrated into 500 µl, and then utilized to infect 50,000 cells in the presence of 5 μg/ml polybrene. Constitutive *ASF1A* overexpressing or knockdown cells were selected using 2 μg/ml puromycin. Sequences for shRNAs are listed blow, shASF1A-1 (5’-CAAUGUGAAGAAUUUGUUU-3’) and shASF1A-2 (5’-GGCAUAUGUUUGUA UUUCA-3’).

### Flow cytometry

K562 and MEG01 cells with *ASF1A* knocked down were seeded in 6-well plates and then treated with dimethyl sulfoxide (DMSO), FLI-06, or IMR-1 for 48 h. A total of 10^6^ cells were harvested, washed twice with PBS, and resuspended in 100 μl PBS containing 20 μl of antibodies against CD61 (BD Pharmingen, USA, CAT#555754) and CD13 (BD Pharmingen, USA, CAT#338425). The cell-antibody mixture was incubated for 30 min in the dark, washed and resuspended with PBS, and then analyzed by flow cytometry using a FACScan (Becton Dickinson, USA).

### RNA-Seq analysis

RNA content was isolated from K562 cells treated with sh-*NC* or sh*ASF1A*-1, and whole transcriptome analysis was performed by RNA sequencing. Sequencing was performed using an Illumina HiSeq 4000 system. Data analysis was performed using the gene-set enrichment analysis (GSEA). The RNA sequencing procedures and experiments were outsourced to KangChen Bio-tech (Shanghai, China).

### Tumor xenograft model

For the xenograft model, six NOD/SCID male mice (Hua Fu Kang Biological Technology, Beijing, China) were treated with 2 Gy radiation. 1 × 10^6^ K562 cells transduced with sh*ASF1A-1* or sh*NC* were subcutaneously injected into the right or left flank of the mice. Tumor growth was monitored every 3 days. All animal procedures were approved by the Qilu Hospital of Shandong University Research Ethics Committee. The animal study was conducted in accordance with the ARRIVE guidelines [22].

### May-Grünwald Giemsa staining

After treating the cells with the indicated conditions, cells were collected and washed with PBS. Cells were mounted on glass slides, and morphological evaluation of differentiation was assessed using a May-Grünwald Giemsa staining kit. Samples were dried at room temperature and observed using a fluorescence inversion microscope system (Olympus, USA).

### Statistical analysis

All experiments were repeated at least three times. The cell lines are applied with three independent lentiviral infections, treated with or without the compounds. Data obtained from biological replicates are presented as mean ± SD. Student’s *t* test and one-way analysis of variance were used to analyze the differences between groups using GraphPad Prism (GraphPad Software, La Jolla, CA, USA). All tests are one sided. A *p* value (*P*) < 0.05, was used as a cut-off for a statistically significant difference.

## Results

### ASF1A suppresses cell differentiation to enhance CML transformation

**Fig. 1:**
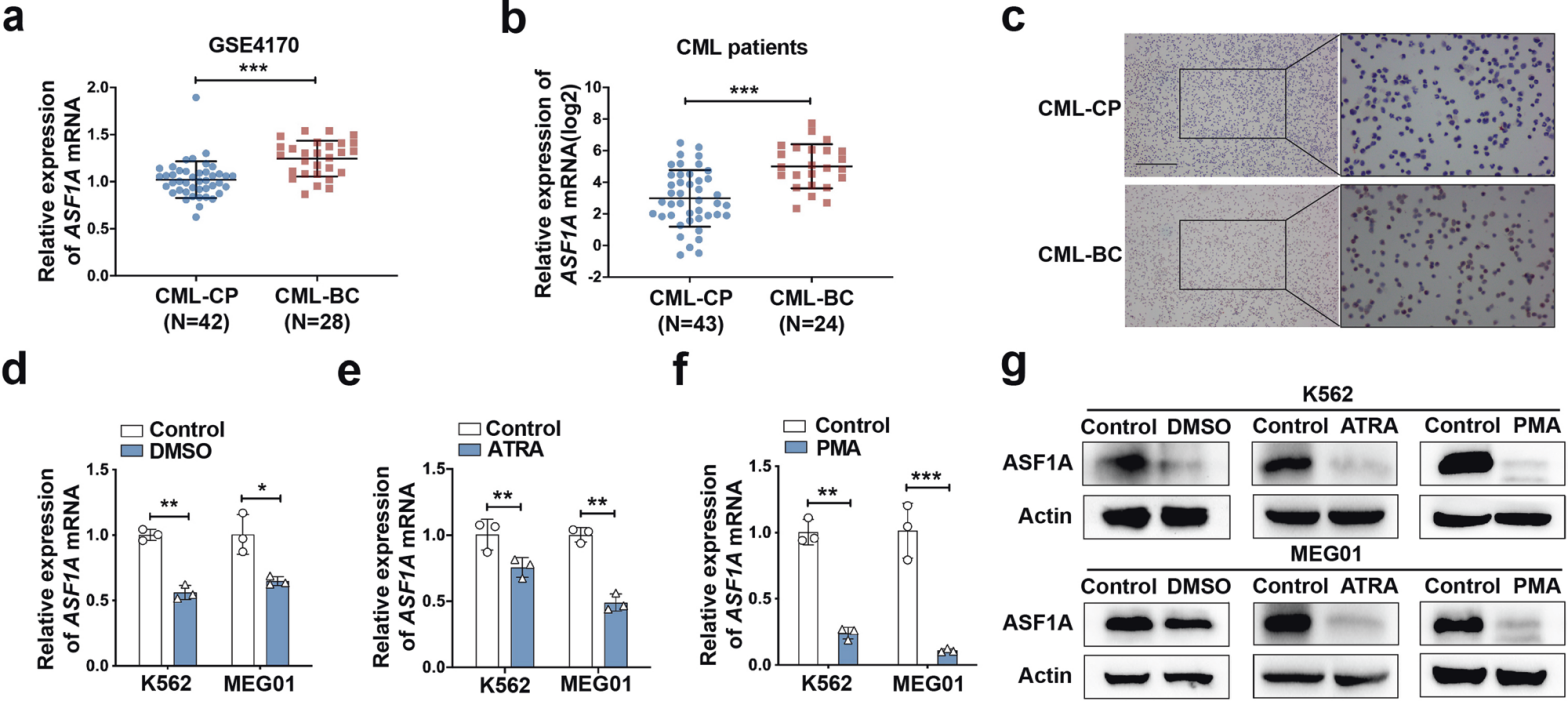
ASF1A was upregulated in CML-BC patients and involved in cell differentiation. **a** Relative expression of *ASF1A* mRNA in CML-CP and CML-BP patients using the GEO database (GSE4170). **b** qRT-PCR analysis of *ASF1A* mRNA levels in CML-CP and CML-BP patients. **c** ICC analysis of ASF1A protein levels. Scale bar, 200 μm. **d**–**f** qRT-PCR analysis of *ASF1A* mRNA levels in K562 and MEG01 cells pretreated with solvent (control) or DMSO/ATRA/PMA. **g** Western blot analysis of ASF1A in K562 and MEG01 cells pretreated with solvent (control) or DMSO/ATRA/PMA. Statistical significance was determined by Student’s *t*-test. Data are shown as mean ± standard deviation (SD). Data are shown as a representative result with three repeats from three independent experiments. The cell lines are applied with three independent lentiviral infections in **d**–**g**. *P < 0.05, **P < 0.01, ***P < 0.001.

To determine the potential role of ASF1 in the BC transition of CML, we first examined its expression in CML-BC and CML-CP patients. In GSE4170, the expression of ASF1A, but not ASF1B, was much higher in CML-BC (*n* = 28) than in CML-CP (*n* = 42) patients (Fig. 1a and Supplementary Fig. S1a). Similarly, we measured the mRNA and protein expression of ASF1A in bone marrow samples from patients with newly diagnosed CML-CP (*n* = 43) and CML-BC (n = 24), and both ASF1A mRNA and protein expressions were enhanced in patients with CML-BC than in those with CML-CP (Fig. 1b, c). In addition, the receiver operating characteristic (ROC) analysis based on the data of both GSE4170 and the clinical samples showed that ASF1A had a statistically significant large area under the curve (Supplementary Fig. S1b, c). Differentiation-arrest is the most common feature for the transformation of CML from CP to BC [3, 5]. We next compared ASF1A expression during different stages of differentiation. DMSO, ATRA, and PMA are different inducers for CML cell differentiation [23], and treatment with these inducers greatly suppressed the mRNA and protein expression of ASF1A in K562 and MEG01 cells (Fig. 1d–g). Overall, these data support the idea that ASF1A is activated and upregulated in CML-BC, thereby suggesting that ASF1A may contribute to CML transformation.

**Fig. 2:**
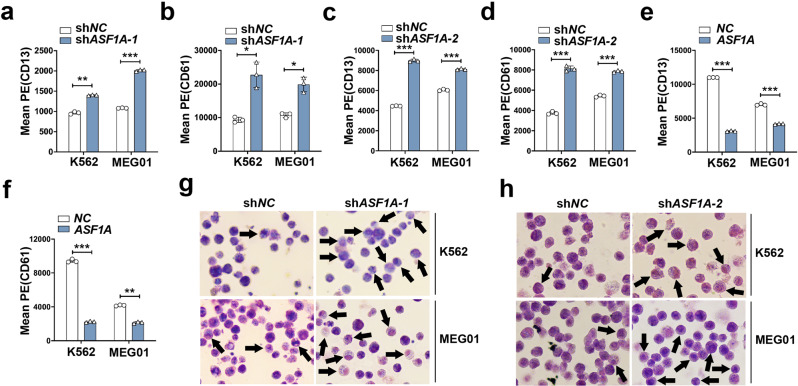
ASF1A suppresses cell differentiation to accelerate CML transformation. **a**–**d** FACS analysis of CD13 and CD61 levels in K562 and MEG01 cells expressing either empty vector (sh*NC*) or sh*ASF1A-1*/sh*ASF1A-2*. **e**, **f** FACS analysis of CD13 and CD61 levels in K562 and MEG01 cells expressing either empty vector (*NC*) or *ASF1A*. **g**, **h** Cell morphological assays assessed by May-Grunwald Giemsa staining of K562 and MEG01 cells expressing either empty vector (sh*NC*) or sh*ASF1A-1*/sh*ASF1A-2*. Arrows indicate cells with matured morphology, which exhibit kidney-shape nucleus and decreased nuclear/cytoplasm ratio. Statistical significance was determined by Student’s *t* test. Data are shown as mean ± standard deviation (SD). Data are shown as a representative result with three repeats from three independent experiments. The cell lines are applied with three independent lentiviral infections in **a**–**h**. *P < 0.05, **P < 0.01, ***P < 0.001.

To clarify the potential role of ASF1A in CML transformation, we generated lentivirus expressing shRNA against *ASF1A* to disrupt endogenous *ASF1A* expression, and then transduced K562 and MEG01 cells (Supplementary Fig. S2a, b). The mRNA expression of ASF1B showed no effect by ASF1A knockdown (Supplementary Fig. S2d, e). We then examined the expression of CD13 and CD61 by flow cytometry, which are indicators of myeloid and megakaryocyte differentiation [24]. *ASF1A* knockdown substantially enhanced CD13 and CD61 expression in both K562 and MEG01 cells (Fig. 2a–d, Supplementary Fig. S2f, h). Moreover, *ASF1A* knockdown induced a more significantly matured appearance of cells with kidney-shaped nuclei and decreased nuclear/cytoplasm ratio (Fig. 2g, h), suggesting that inhibiting *ASF1A* expression promotes morphological differentiation. Conversely, we also generated lentivirus overexpressing *ASF1A* to elevate endogenous *ASF1A* expression, and then transduced K562 and MEG01 cells (Supplementary Fig. S2c). Flow cytometry showed that *ASF1A* overexpression substantially inhibited CD13 and CD61 expression in both K562 and MEG01 cells (Fig. 2e, f, Supplementary Fig. S2h). Taken together, these data suggest that ASF1A suppresses the differentiation of CML cells and contributes to the transformation of CML from CP to BC.

### ASF1A mediates differentiation arrest by enhancing Notch signaling activation

**Fig. 3:**
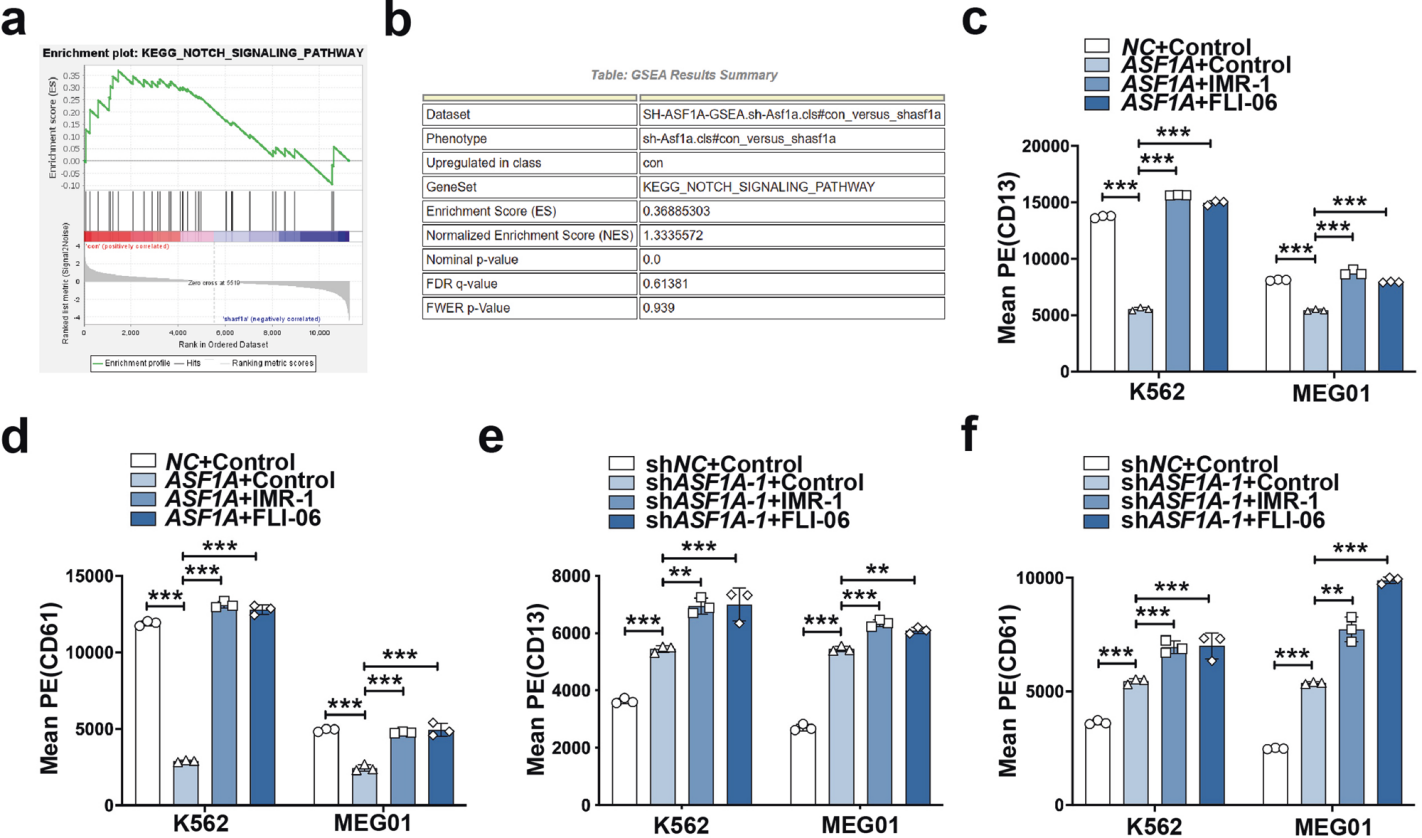
ASF1A enhanced CML transformation via Notch signaling. **a**, **b** Gene-set-enrichment analysis was performed using transcriptomes of K562 cells expressing empty vector (sh*NC*) vs sh*ASF1A-1* (**a**). The specific properties of the plot in **b**. **c**, **d** FACS analysis of CD13 and CD61 levels in K562 and MEG01 cells expressing either empty vector (*NC*) or *ASF1A*, treated with control, IMR-1 (20 μM) or FLI-06 (5 μM) for 48 h. **e**, **f** FACS analysis of CD13 and CD61 levels in K562 and MEG01 cells expressing either empty vector (sh*NC*) or sh*ASF1A-1*, treated with control, IMR-1 (20 μM) or FLI-06 (5 μM) for 48 h. Statistical significance was determined by One-way ANOVA. Data are shown as mean ± standard deviation (SD). Data are shown as a representative result with three repeats from three independent experiments. The cell lines are applied with three independent lentiviral infections and then treated with the compounds in **c**–**f**. **P < 0.01, ***P < 0.001.

**Fig. 4:**
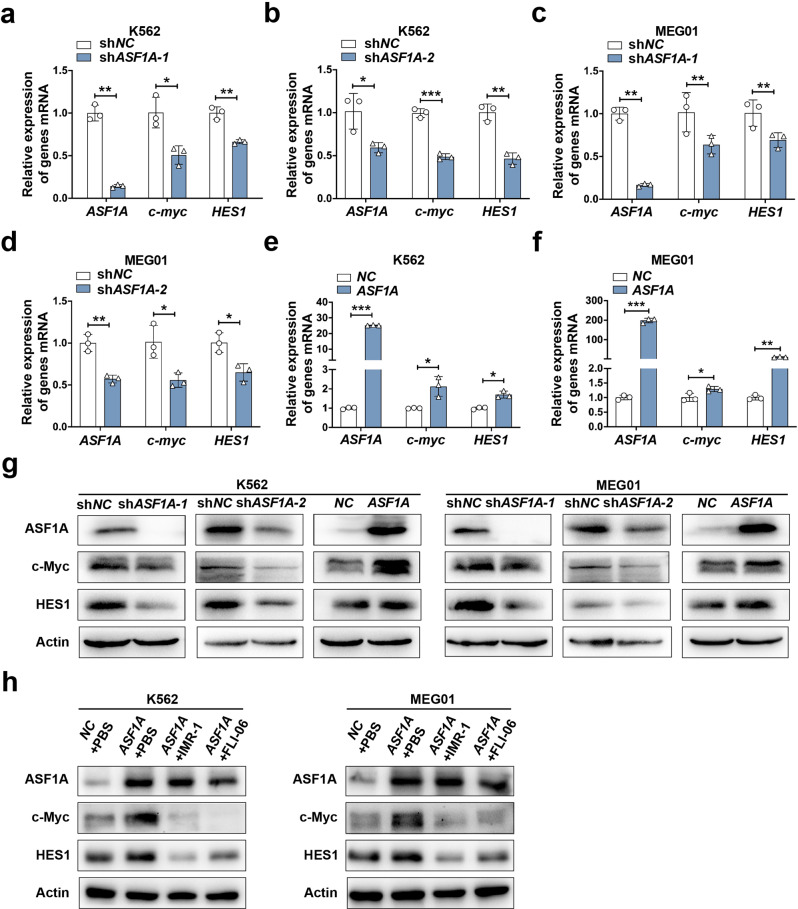
ASF1A promoted Notch signaling activation. **a**, **b** qRT-PCR analysis of *ASF1A*, *c-Myc*, and *HES1* mRNA levels in K562 cells expressing empty vector (shNC) or shASF1A-1/shASF1A-2. **c**, **d** qRT-PCR analysis of *ASF1A*, *c-Myc*, and *HES1* mRNA levels in MEG01 cells expressing empty vector (shNC) or shASF1A-1/shASF1A-2. **e**, **f** qRT-PCR analysis of *ASF1A*, *c-Myc*, and *HES1* mRNA levels in K562 and MEG01 cells expressing either empty vector (NC) or ASF1A. **g** Western blot analysis of ASF1A, c-Myc, and HES1 in K562 and MEG01 cells transfected as indicated. Statistical significance was determined by Student’s *t* test. Data are shown as mean ± standard deviation (SD). Data are shown as a representative result with three repeats from three independent experiments. The cell lines are applied with three independent lentiviral infections in **a**–**g**. *P < 0.05, **P < 0.01, ***P < 0.001. **h** Western blot analysis of ASF1A, c-Myc, and HES1 in K562 and MEG01 expressing either empty vector (NC) or ASF1A, treated with control, IMR-1 (20 μM) or FLI-06 (5 μM) for 48 h. Data are shown as a representative result with three repeats from three independent experiments. The cell lines are applied with three independent lentiviral infections and then treated with the compounds in **h**.

To clarify the mechanism by which ASF1A attenuates cell differentiation in CML, we next performed RNA-seq analyses of K562 cells expressing shASF1A-1 or empty vector. KEGG analysis is applied to show that ASF1A knockdown could enrich a few important pathways (shown in Supplementary Table 3 and Supplementary Fig. S3). Surprisingly, GSEA analysis showed that the Notch signaling pathway was enriched in the control group compared to the *ASF1A* knockdown group (Fig. 3a, b). We next investigated the function of ASF1A in Notch signaling activation. The small molecules IMR-1 and FLI-06 are different inhibitors of the Notch signaling pathway [25, 26]. The ASF1A overexpression-mediated decrease in CD13 and CD61 expression was completely reversed by IMR-1 and FLI-06 treatment in both K562 and MEG01 cells (Fig. 3c, d and Supplementary Fig. S4a–d). Moreover, treatment with IMR-1 and FLI-06 further promoted *ASF1A* knockdown-mediated increase in CD13 and CD61 expression (Fig. 3e, f and Supplementary Fig. S4e–h). *c-Myc* and *HES1* are important Notch target genes implicated in hematopoietic cell differentiation [11, 27]. Consequently, *ASF1A* knockdown inhibited the mRNA and protein expression of c-Myc and HES1 in both K562 and MEG01 cells (Fig. 4a–d, g). Conversely, the mRNA and protein expression of c-Myc and HES1 were upregulated by *ASF1A* overexpression (Fig. 4e–g). The NOTCH inhibitors, including IMR-1 and FLI-06, rescue the ASF1A overexpression-induced enhancement of c-Myc and HES1 expression (Fig. 4h). Overall, these data suggest that ASF1A mediates differentiation arrest by enhancing Notch signaling activation.

### ASF1A cooperates with RBPJ activates Notch signaling by promoting H3K56ac

**Fig. 5:**
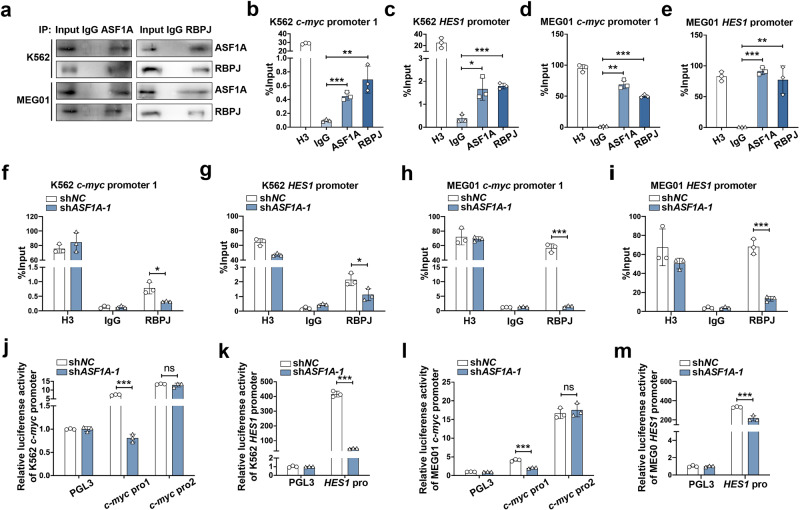
ASF1A cooperates with RBPJ activates Notch signaling. **a** Co-immunoprecipitation analysis of the interaction between ASF1A and RBPJ in K562 and MEG01 cells. **b**–**e** Enrichment of ASF1A and RBPJ at *c-Myc* (**b**, **d**) and *HES1* (**c**, **e**) promoters in K562 and MEG01 cells. **f**–**i** RBPJ enrichment at *c-Myc* (**f**, **h**) and *HES1* (**g**, **i**) promoters in K562 and MEG01 cells expressing empty vector (sh*NC*) or sh*ASF1A-1*. Histone H3 and IgG antibodies were used as positive and negative controls, respectively. **j**–**m** Luciferase activity analysis of *c-Myc* (**j**, **l**) and *HES1* (**k**, **m**) activation in K562 and MEG01 cells transfected with empty vector (sh*NC*) or sh*ASF1A-1*. Luciferase activity was determined 48 hours after transfection, and normalized according to Renilla luciferase activity. *P* values in **b**–**e** were determined by one-way ANOVA, and *P* values in **f**–**m** were calculated using Student’s *t* test. Data are shown as mean ± standard deviation (SD). Data are shown as a representative result with three repeats from three independent experiments. The cell lines are applied with three independent lentiviral infections in **b**–**m**. *P < 0.05, **P < 0.01, ***P < 0.001.

Upon Notch signaling activation, the key transcription RBPJ recognizes the NICD and subsequently initiates the transcription of Notch target genes [7, 8]. To investigate whether ASF1A controls RBPJ activity, we first examined the association between ASF1A and RBPJ. ASF1A coprecipitated with RBPJ in both K562 and MEG01 cells (Fig. 5a). It has been established that RBPJ specifically binds to the TGGGAA motif, which is present in the promoter regions of multiple Notch target genes, including *c-Myc* and *HES1* [28, 29]. Next, we performed ChIP assay and found that the ASF1A/RBPJ complex could bind to the promoter region of *c-Myc* and *HES1* in both K562 and MEG01 cells (Fig. 5b–e and Supplementary Fig. S5a), while promoter 2 of *c-Myc* as negative control did not bind to the ASF1A/RBPJ complex (Supplementary Fig. S5b, c). Next, we examined the role of ASF1A in the Notch transcriptional complex. *ASF1A* knockdown inhibited RBPJ binding to the promoter region of *c-Myc* and *HES1* in both K562 and MEG01 cells (Fig. 5f–i). Moreover, *ASF1A* knockdown suppressed the promoter activation of *c-Myc* promoter 1 and *HES1*, while no significant change was observed in *c-Myc* promoter 2 (Fig. 5j–m). These results support that ASF1A is a novel coactivator of the Notch transcriptional complex with RBPJ to enhance Notch signaling activation.

**Fig. 6:**
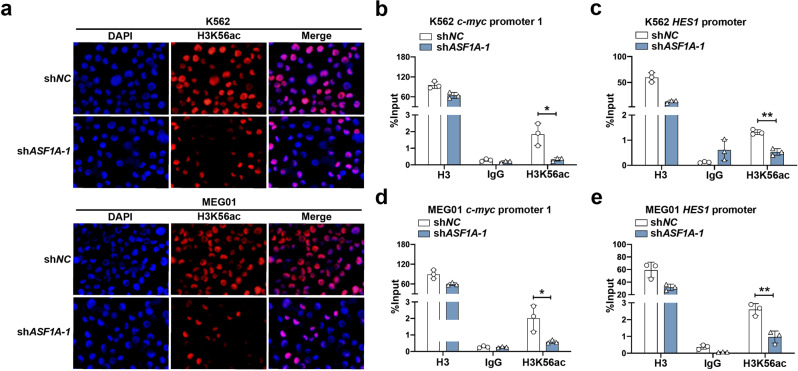
ASF1A licenses H3K56ac modification in the promoter regions of Notch target genes. **a** IF staining of histone H3K56 acetylation in K562 and MEG01 cells expressing empty vector (sh*NC*) or sh*ASF1A-1*. **b**–**e** Enrichment of H3K56ac at *c-Myc* (**b**, **d**) and *HES1* (**c**, **e**) promoters in K562 and MEG01 cells expressing empty vector (sh*NC*) or sh*ASF1A-1*. Histone H3 and IgG antibodies were used as positive and negative controls, respectively. Statistical significance was determined by Student’s *t* test. Data are shown as mean ± standard deviation (SD). Data are shown as a representative result with three repeats from three independent experiments. The cell lines are applied with three independent lentiviral infections in **a**–**e**. *P < 0.05, **P < 0.01.

Histone modification is an essential mechanism for regulating gene expression and shaping functional chromatin states [30]. ASF1A is specifically required for the H3K56ac, which affects gene expression and promotes transcription factors that bind to the target gene promoters [20, 21, 31]. The level of H3K56ac was suppressed by *ASF1A* knockdown in both K562 and MEG01 cells (Fig. 6a). Furthermore, *ASF1A* knockdown significantly decreased the level of H3K56ac at the promoters of *c-Myc* and *HES1* (Fig. 6b–e). Overall, these data suggest that ASF1A acts as a coactivator with RBPJ to induce H3K56ac modification in the promoter regions of Notch target genes, thereby enhancing RPBJ binding to these promoter regions.

### ASF1A promotes the development of CML in vivo

**Fig. 7:**
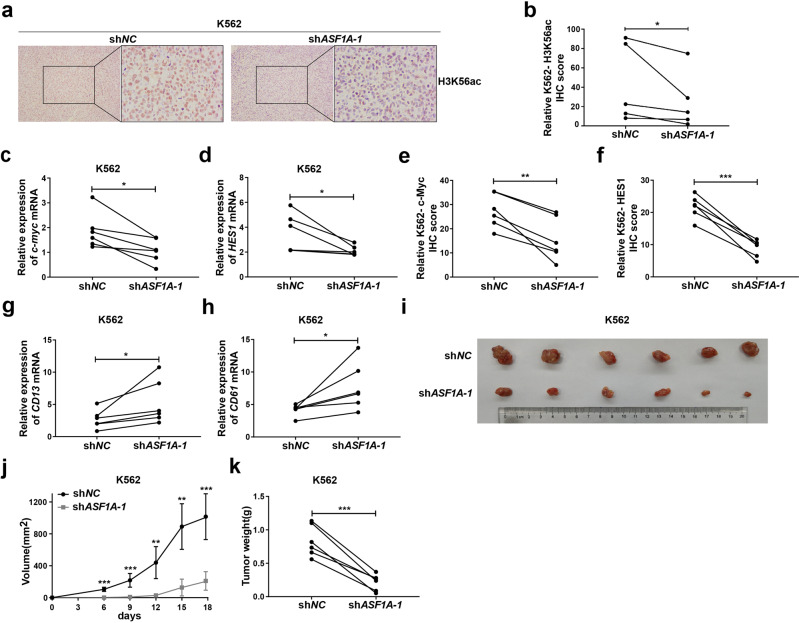
ASF1A promotes the development of CML in vivo. **a**–**h** NOD-SCID mice were injected subcutaneously with K562 cells expressing empty vector (sh*NC*) or sh*ASF1A-1*. The protein level of H3K56Ac were analyzed by IHC (**a**, **b**). The mRNA and protein levels of c-Myc (**c**, **e**), HES1 (**d**, **f**), CD13 (**g**), and CD61 (**h**) were analyzed by qRT-PCR and IHC. β2-M expression was used for normalization. **i**–**k** Primary tumor gross appearance (**i**), tumor growth curve (**j**), and tumor weight analysis (**k**) of NOD-SCID mice injected subcutaneously with K562 cells expressing empty vector (sh*NC*) or sh*ASF1A-1*. Statistical significance was determined by Student’s *t* test. Data are shown as mean ± standard deviation (SD). Data are presented as the mean ± SD of five biologically independent animals in **a**, **b**. Data are shown as a representative result with three repeats from three independent experiments. The cell lines are applied with three independent lentiviral infections and then treated with the compounds in **c**–**h**. Data are presented as the mean ± SD of six biologically independent animals in **i**–**k**. *P < 0.05, **P < 0.01, ***P < 0.001.

To investigate the physiological and pathological relevance of ASF1A in the development of CML in vivo, we established a xenograft tumor model by injecting K562 cells transduced with shRNA against *ASF1A* (or scrambled shRNA) subcutaneously into NOD-SCID mice. The knockdown efficiency was confirmed prior to beginning the xenograft model (Supplementary Fig. S6a–c). ASF1A knockdown reduced H3K56ac in vivo by a xenograft tumor model, suggesting that ASF1A enhanced the development of CML via H3K56ac in vivo (Fig. 7a, b). The mRNA and protein expression of c-Myc and HES1 were significantly downregulated by *ASF1A* knockdown (Fig. 7c–f and Supplementary Fig. S6c). The differentiation-related markers CD13 and CD61 were increased after *ASF1A* knockdown (Fig. 7g, h). Furthermore, we observed that the tumors derived from *ASF1A* knockdown group were much smaller (Fig. 7i), and the tumor size and weight were reduced in the *ASF1A* knockdown groups (Fig. 7j–k). Overall, these data suggest that ASF1A is an essential activator of Notch signaling and is required for activation and development of CML in vivo (Supplementary Fig. S7).

## Discussion

As the final phase of CML, BC remains a major challenge in clinical management [32]. Tyrosine kinase inhibitors (TKIs), which target the BCR-ABL fusion protein, have transformed long-term outcomes in patients with CML-CP. Once the disease progresses to CML-BC, it manifests more aggressive pathology and is highly resistant to conventional chemotherapy and TKI treatment. However, the molecular mechanism underlying the transformation to CML-BC remains unclear. In the present study, we defined ASF1A as an essential activator driving the transformation to CML-BC by mediating cell differentiation arrest.

ASF1A is a histone H3-H4 chaperone with putative oncogenic activities and has been reported to be overexpressed in multiple malignancies [33–35]. Recently, ASF1A has been reported to inhibit the sensitization of *Kras*-mutant lung adenocarcinoma to anti-PD-1 treatment [36]. In addition, ASF1A can enhance the progression of gastrointestinal cancer by potentiating the transcription of β-catenin target genes [34]. These studies suggest that ASF1A could serve as a prognostic factor and a potential target in numerous types of cancer. Our results show that ASF1A is aberrantly upregulated in bone marrow samples from patients with CML-BC compared to newly diagnosed CML-CP, and ASF1A inhibits differentiation of leukemia cells into granulocytes and macrophages, thereby driving the transformation to CML-BC.

Although Notch signaling plays a central role in development and homeostasis, dysregulated Notch signaling has been recognized as an important driver of various malignancies [7, 8]. Notch signaling has been reported to be widely and constitutively overactivated in CML, and its activation is elevated in CML-BC compared to CML-CP [9]. *BCR-ABL* silencing suppresses Notch signaling activation by reducing *NOTCH1* expression [37]. The c-Myc and HES1 are known to be downstream effector molecules of the Notch pathway, and their oncogenic role in the transformation of CML in BC is well-established [8, 10, 11, 38, 39]. Therefore, understanding how Notch signaling is dysregulated in CML is important to understand CML-BC transformation. Our results show that ASF1A is aberrantly increased in CML-BC, which acts as a coactivator of the Notch transcriptional complex with RBPJ, to enhance Notch signaling activation, and subsequently induce differentiation arrest to drive the transformation to CML-BC.

Histone modifications play essential roles in regulating gene expression and shaping functional chromatin states [30]. H3K56ac was first detected in yeast, and in contrast to the abundant H3K56ac in yeast and *Drosophila*, H3K56ac marks less than 1% of total H3 in human cells [40–42]. H3K56ac has been reported to be required for the development of embryonic stem cells and maintenance of genome integrity in normal physiological conditions [40, 41], affects gene expression, and promotes transcription factors that bind to the target gene promoters [21, 31]. Moreover, H3K56ac in human cells is not as global as yeast, but is gene-specific [41]. Thus, aberrant modifications by H3K56ac may drive malignant diseases. ASF1A is specifically required for H3K56ac by cooperating with histone acetyltransferase p300, and H3K56ac is increased in multiple types of cancer, correlating with increased *ASF1A* expression [20]. Rapid changes in H3K56ac at Notch-regulated enhancers were detected during its activation in *Drosophila* [43]. Our results suggest that ASF1A modifies H3K56ac in the promoter regions of Notch target genes and subsequently enhances RBPJ binding to these promoter regions, thereby promoting Notch target gene expression.

In summary, our work identifies ASF1A as an essential activator contributor to CML transformation by aberrantly mediating Notch signaling activation. This suggests that targeting ASF1A might be a promising therapeutic approach and a biomarker to detect phase progression in CML patients. Finally, dysregulated Notch signaling is essential for driving various malignancies, raising the possibility that ASF1A may also be relevant in other malignancies.

## Supplementary information

supplemental figures and legend

Supplemental Figure.S1

Supplemental Figure.S2

Supplemental Figure.S3

Supplemental Figure.S4

Supplemental Figure.S5

Supplemental Figure.S6

Supplemental Figure.S7

checklist

## Data Availability

All data supporting the findings of this study are available from the corresponding author upon reasonable request.
