# Supplementary material for: Histone chaperone ASF1A accelerates chronic myeloid leukemia blast crisis by activating Notch signaling
Source: Cell Death Dis. 2022 Oct 3;13(10):842. doi: 10.1038/s41419-022-05234-5 (PMC9527247; doi:10.1038/s41419-022-05234-5)
Supplement: Supplementary file 1 — supplemental figures and legend [file 41419_2022_5234_MOESM1_ESM.docx]

**
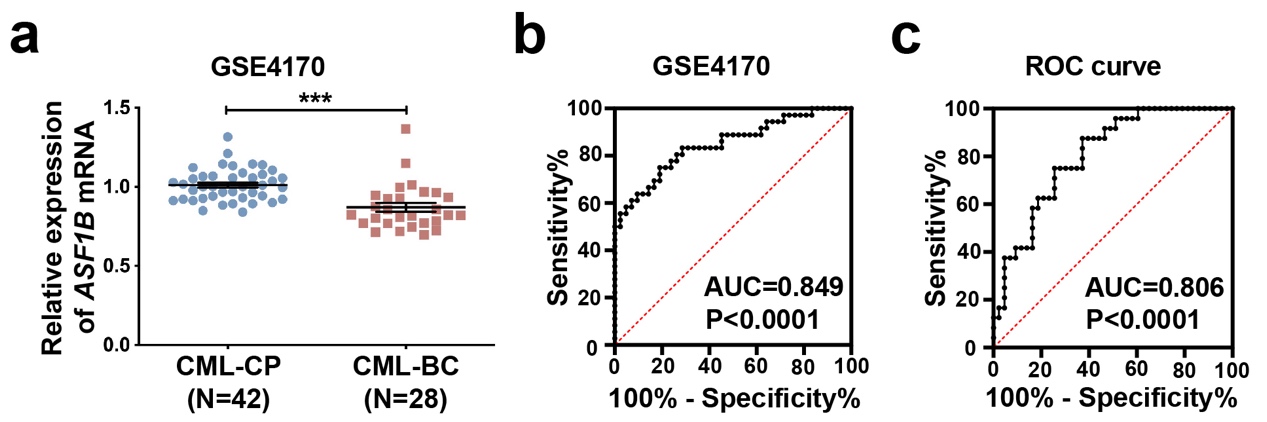
**

**Fig. S1. The receiver operating characteristic (ROC) analysis of ASF1A.**

**a** Relative expression of *ASF1B* mRNA in CML-CP and CML-BP patients using the GEO database (GSE4170). **b** ROC analysis of ASF1A based on the data of GSE4170. **c** ROC analysis of ASF1A based on the clinical samples. Statistical significance was determined by Student’s *t*-test. Data are shown as mean ± standard deviation (SD). ****P*< 0.001.


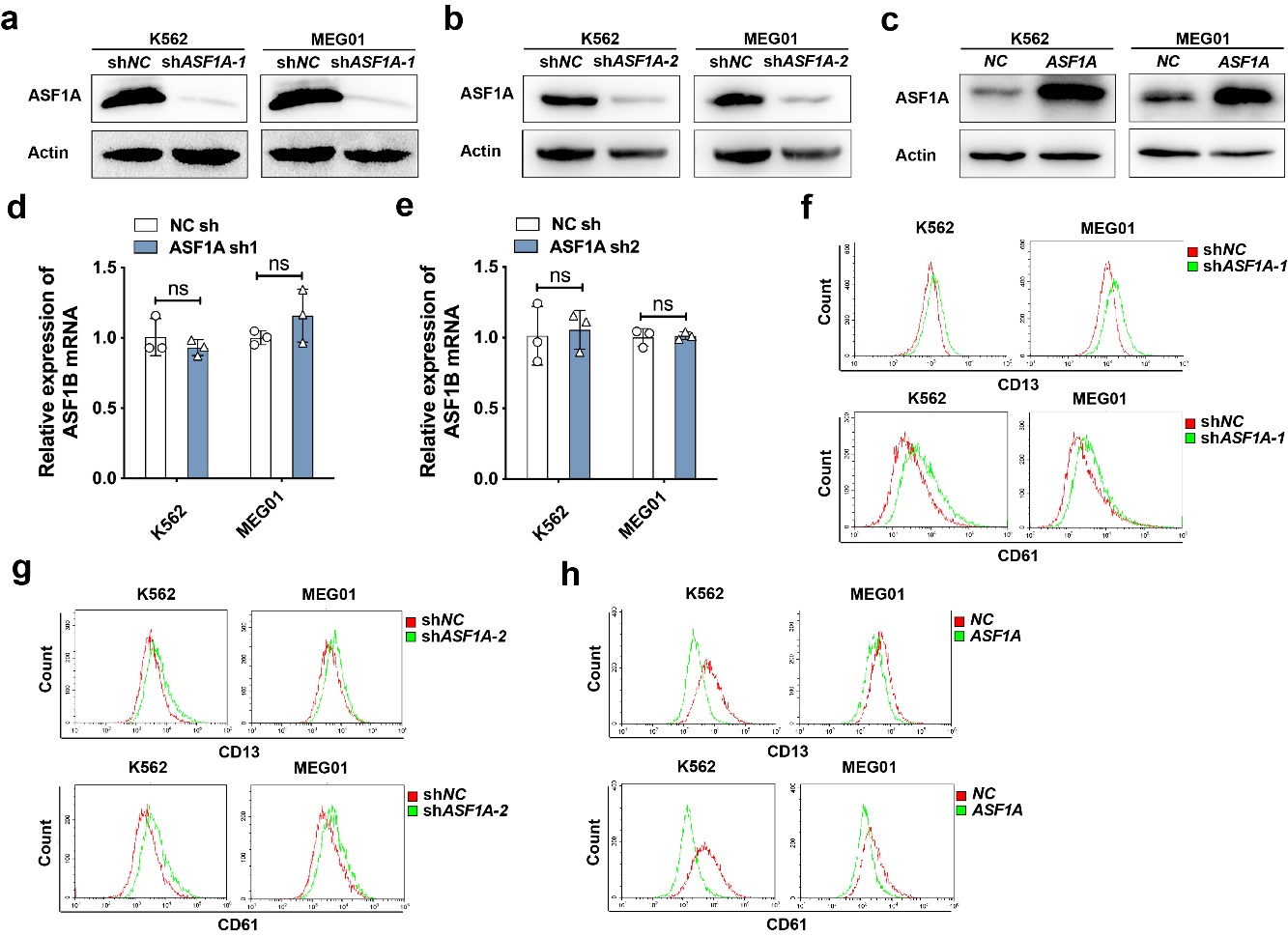


**Fig. S2. ASF1A inhibited cell differentiation to enhance CML transformation.**

**a-b** Western blot analysis of ASF1A in K562 and MEG01 cells expressing either empty vector (sh*NC*) or sh*ASF1A-1* **(a)**/ sh*ASF1A-2* **(b)**. **c** Western blot analysis of ASF1A in K562 and MEG01 cells expressing either empty vector (*NC*) or *ASF1A.* **d-e** qRT-PCR analysis of ASF1B mRNA levels in K562 and MEG01 cells expressing empty vector (shNC) or shASF1A-1/ shASF1A-2. **f-g** FACS analysis of CD13 and CD61 levels in K562 and MEG01 cells expressing either empty vector (sh*NC*) or sh*ASF1A-1* **(f)**/ sh*ASF1A-2* **(g)**. **h** FACS analysis of CD13 and CD61 levels in K562 and MEG01 cells expressing either empty vector (*NC*) or *ASF1A.* Data are shown as a representative result with 3 repeats from three independent experiments. The cell lines are applied with 3 independent lentiviral infections in **a-h.** ns P>0.05.

**Fig.S3. KEGG analysis of genes signiﬁcantly changed in cells expressing sh-ASF1A or sh-NC.**

RNA-seq analyses of K562 cells constitutively expressing sh-ASF1A or sh-NC (negative control) were performed. A total of 536 up-regulated genes and 347 down-regulated genes has been identified (*P*<0.05, |log^2^FC|>1.5). Based on the significant number of differential genes detected, we further performed KEGG analyses. Significant pathways are enriched of genes signiﬁcantly changed.


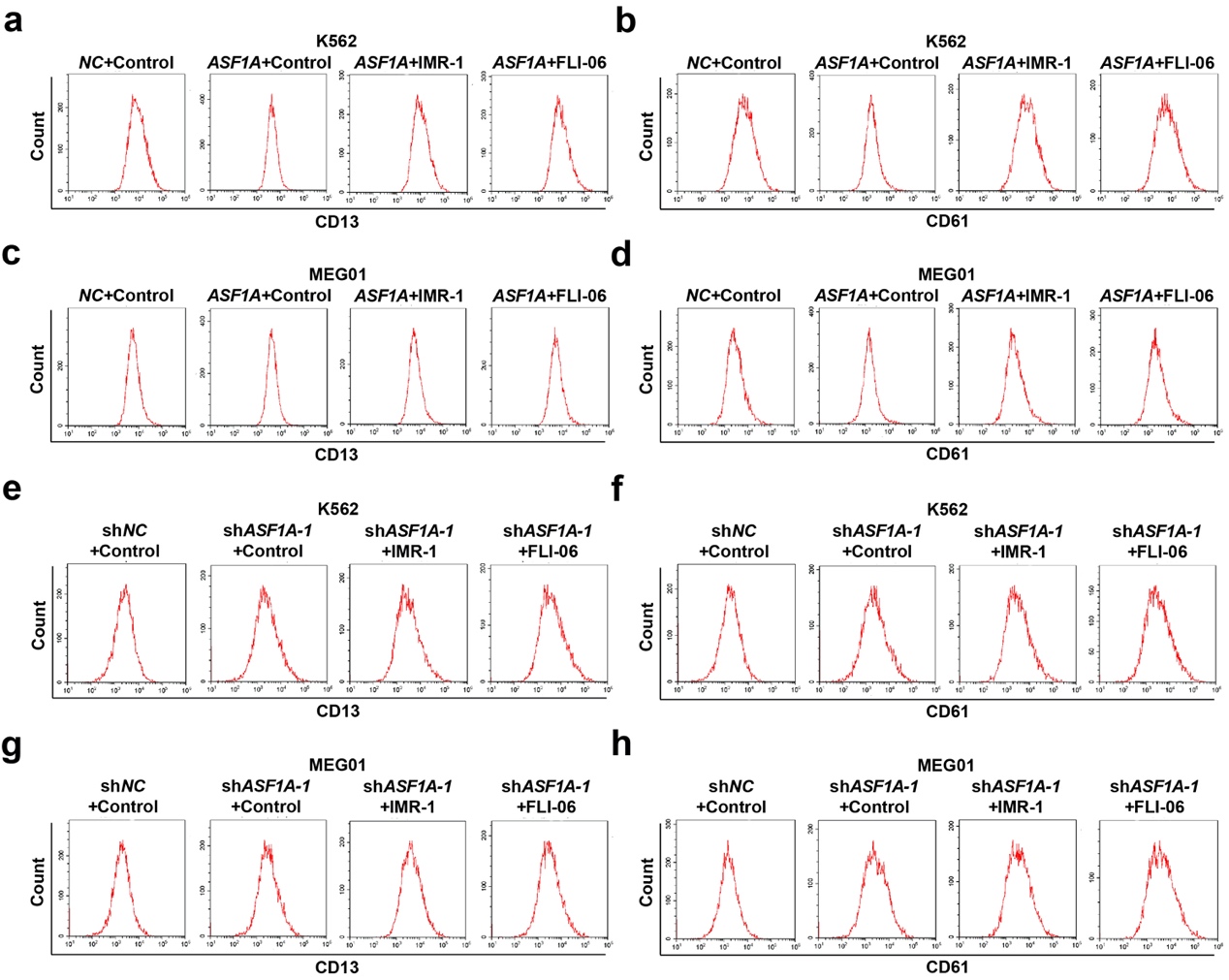


**Fig. S4. ASF1A enhanced Notch signaling activation.**

**a-d** FACS analysis of CD13 and CD61 levels in K562 and MEG01 cells expressing either empty vector (*NC*) or *ASF1A*, treated with control, IMR-1 (20uM) or FLI-06 (5uM) for 48h. **e-h** FACS analysis of CD13 and CD61 levels in K562 and MEG01 cells expressing either empty vector (sh*NC*) or sh*ASF1A-1*, treated with control, IMR-1 (20uM) or FLI-06 (5uM) for 48h. Data are shown as a representative result with 3 repeats from three independent experiments.


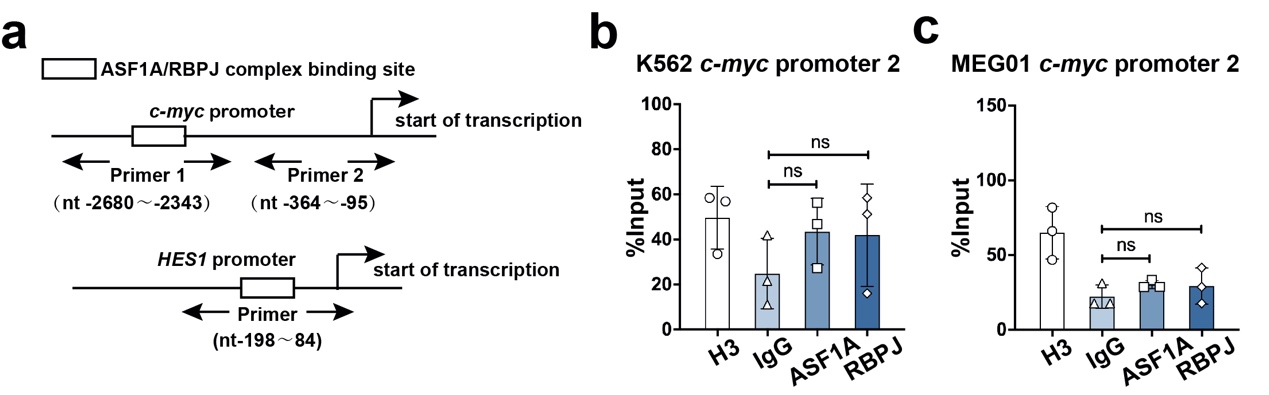


**Fig. S5. ASF1A cooperates with RBPJ activates Notch signaling.**

**a** Illustration of RBPJ/ASF1A complex binding motifs in c-Myc and HES1 promoters. **b-c** Enrichment of ASF1A and RBPJ at *c-myc* promoters in K562 and MEG01 cells. Histone H3 and IgG antibodies were used as positive and negative controls, respectively. Statistical significance was determined by One-way ANOVA. Data are shown as mean ± standard deviation (SD). Data are shown as a representative result with 3 repeats from three independent experiments in **b-c**.


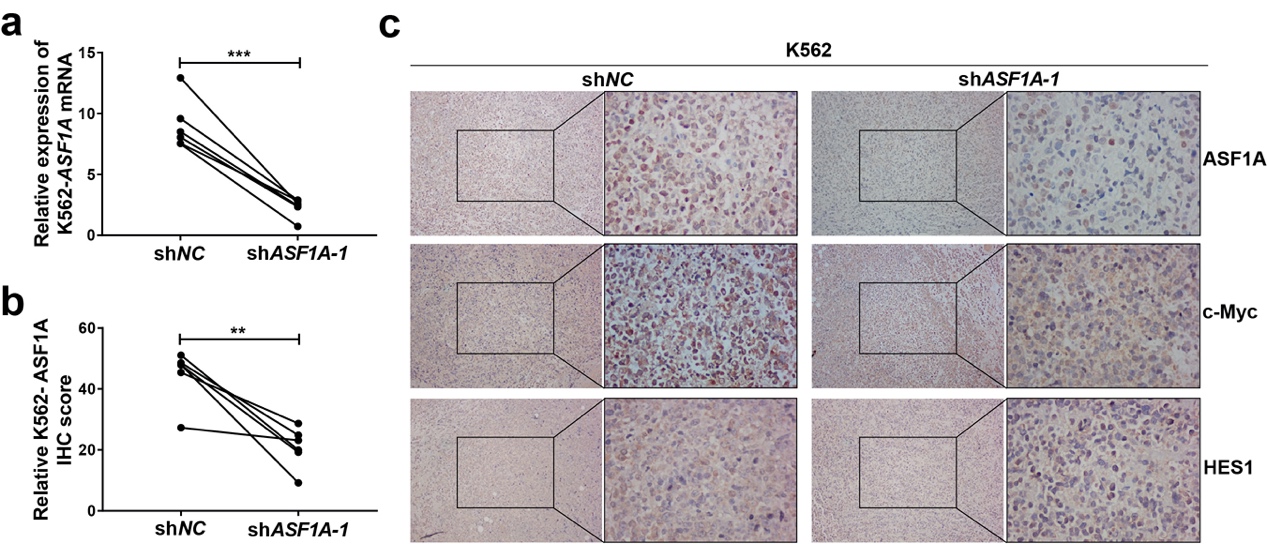


**Fig. S6. The knockdown efficiency of *ASF1A* in the xenograft tumor model.**

NOD-SCID mice were injected subcutaneously with K562 cells expressing empty vector (sh*NC*) (N=6) or sh*ASF1A-1* (N=6)*.* **a** The mRNA level of *ASF1A* was analyzed by qRT-PCR. **b-c** The protein level of ASF1A was analyzed by IHC (**b**) and was evaluated by the IHC score (**c**). The protein levels of c-Myc and HES1 were analyzed by IHC (**c**). Statistical significance was determined by Student’s *t*-test. Data are presented as the mean ± SD of 6 biologically independent animals in **a-c.** ***P*< 0.01, ****P*< 0.001.


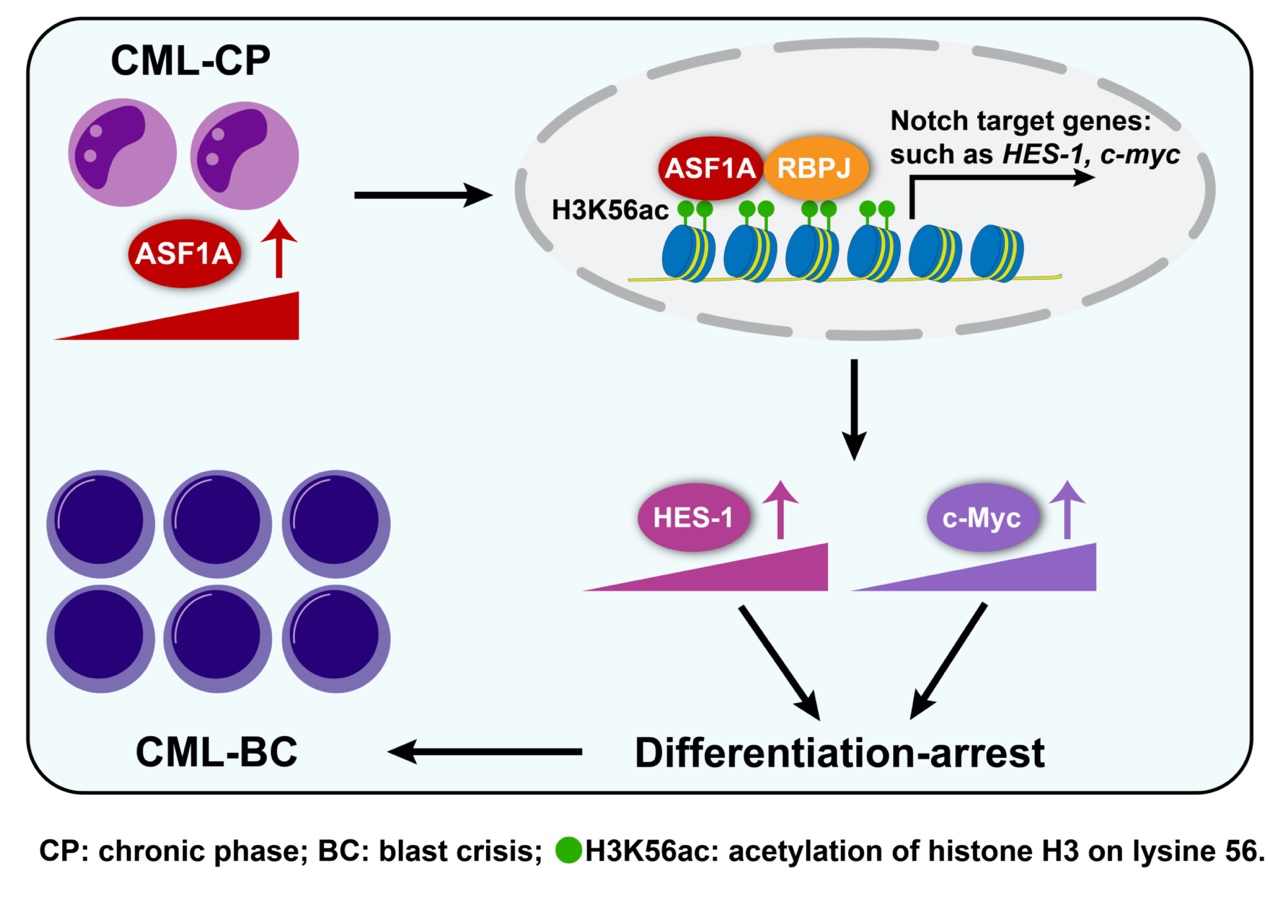


**Fig. S7. Schematic representation of the role of ASF1A in CML blast crisis.**

ASF1A is aberrantly increased in CML-BC patients, which acts as a co-activator of the Notch transcriptional complex with RBPJ that induces H3K56ac modification in the promoter regions of Notch target genes (i.e. *HES-1*, *c-myc*), and subsequently enhances RPBJ binding to these promoter regions, thereby promoting Notch target gene expression to mediate differentiation arrest in CML cells. Consequently, ASF1A as an essential activator contributes to CML transformation by aberrantly mediating Notch signaling activation.

**Supplementary table 1 Clinical characteristics of CML patients.**

| **Characteristic** | | **Patients (n=67)** |
| --- | --- | --- |
| Progression | CP | 43 |
|  | BC | 24 |
| Gender | Male | 36 |
|  | Female | 31 |
| Age (years) | Range | 20-70 |
|  | Medium | 45 |
| WBC, ×10^9^/L | Range | 2.71-370 |
|  | Medium | 72.3 |

**Supplementary table 2 The sequences of primers.**

| **Primers** | | **sequences** |
| --- | --- | --- |
| Actin | Forward | 5’-AGTTGCGTTACACCCTTTCTTG-3’ |
|  | Reverse | 5’-CACCTTCACCGTTCCAGTTTT-3’ |
| HES1 | Forward | 5’-TCAACACGACACCGGATAAAC-3’ |
|  | Reverse | 5’-GCCGCGAGCTATCTTTCTTCA-3’ |
| β2-M | Forward | 5’-GAATTGCTATGTGTCTGGGT-3’ |
|  | Reverse | 5’-CATCTTCAAACCTCCATGATG-3’ |
| c-Myc | Forward | 5’-TACCCTCTCAACGACA  GCAGCTCGCCCAACTCCT-3’ |
|  | Reverse | 5’-TCTTGACATTCTCCTC  GGTGTCCGAGGACCT-3’ |
| p21 | Forward | 5’-TGTCCGTCAGAACCCATGC-3’ |
|  | Reverse | 5’-AAAGTCGAAGTTCCATCGCTC-3’ |
| ASF1A | Forward | 5’-CAGATGCAGATGCAGTAGGC-3’ |
|  | Reverse | 5’-CCTGGGATTAGATGCCAAAA-3’ |
| CD13 | Forward | 5’-GACGCTGAGACCGTACCTC-3’ |
|  | Reverse | 5’-TCAGTCTTGTCAATGTCGGGG-3’ |
| CD61 | Forward | 5’-CATGAAGGATGATCTGTGGAGC-3’ |
|  | Reverse | 5’-AATCCGCAGGTTACTGGTGAG-3’ |
| HES1-CHIP | Forward | 5’-GGCATAAAGAGCAAAGACCCTG-3’ |
|  | Reverse | 5’-CTCGTGTGAAACTTCCCAAACT-3’ |
| c-Myc pro1-  CHIP | Forward | 5’-CATGCCCTACACTGGACTGG-3’ |
|  | Reverse | 5’-TACGGGGCAAAGAATCCCTG-3’ |
| c-Myc pro2-  CHIP | Forward | 5’-TGATTTCTCCCAAACCCG-3’ |
|  | Reverse | 5’-CCAAAGCAGCAGATACCG-3’ |

**Supplementary table 3 pathways are enriched**

|  | logFC | P.Value |
| --- | --- | --- |
| RIBOSOME | -0.71703 | 0.000118 |
| DNA_REPLICATION | 0.701694 | 0.000164 |
| PRIMARY_IMMUNODEFICIENCY | -0.59776 | 0.001301 |
| PROTEASOME | 0.574182 | 0.002003 |
| OLFACTORY_TRANSDUCTION | 0.545233 | 0.003336 |
| GLYCINE_SERINE_AND_THREONINE_METABOLISM | -0.54337 | 0.003444 |
| SELENOAMINO_ACID_METABOLISM | -0.53391 | 0.004047 |
| STEROID_HORMONE_BIOSYNTHESIS | 0.526535 | 0.004583 |
| MISMATCH_REPAIR | 0.506641 | 0.006363 |
| CYSTEINE_AND_METHIONINE_METABOLISM | -0.5024 | 0.006815 |
| VASOPRESSIN_REGULATED_WATER_REABSORPTION | 0.484368 | 0.009077 |
| PROXIMAL_TUBULE_BICARBONATE_RECLAMATION | 0.453737 | 0.01449 |
| FRUCTOSE_AND_MANNOSE_METABOLISM | 0.435098 | 0.019033 |
| AMINO_SUGAR_AND_NUCLEOTIDE_SUGAR_METABOLISM | 0.429213 | 0.020707 |
| GLYCOSAMINOGLYCAN_BIOSYNTHESIS_KERATAN_SULFATE | -0.42809 | 0.021041 |
| GLYCOSPHINGOLIPID_BIOSYNTHESIS_LACTO_AND_NEOLACTO_SERIES | -0.4235 | 0.022452 |
| REGULATION_OF_AUTOPHAGY | 0.419611 | 0.023714 |
| GAP_JUNCTION | 0.408237 | 0.027761 |
| HEDGEHOG_SIGNALING_PATHWAY | -0.40347 | 0.029627 |
| NOTCH_SIGNALING_PATHWAY | -0.39884 | 0.031542 |
| NUCLEOTIDE_EXCISION_REPAIR | 0.395681 | 0.032908 |
| HEMATOPOIETIC_CELL_LINEAGE | 0.379479 | 0.040743 |
| INOSITOL_PHOSPHATE_METABOLISM | -0.37118 | 0.045336 |
| GLYCOSAMINOGLYCAN_BIOSYNTHESIS_HEPARAN_SULFATE | -0.37021 | 0.045899 |
| CELL_CYCLE | 0.365644 | 0.048637 |
| PANTOTHENATE_AND_COA_BIOSYNTHESIS | -0.34769 | 0.060764 |
| PORPHYRIN_AND_CHLOROPHYLL_METABOLISM | 0.344978 | 0.062799 |
| VALINE_LEUCINE_AND_ISOLEUCINE_DEGRADATION | -0.34244 | 0.064754 |
| NON_SMALL_CELL_LUNG_CANCER | 0.325589 | 0.079039 |
| MELANOMA | 0.32353 | 0.080949 |
| BASE_EXCISION_REPAIR | 0.32203 | 0.082364 |
| NITROGEN_METABOLISM | 0.316483 | 0.087771 |
| BLADDER_CANCER | 0.313215 | 0.091088 |
| ALDOSTERONE_REGULATED_SODIUM_REABSORPTION | 0.307664 | 0.096954 |
| BUTANOATE_METABOLISM | 0.300826 | 0.104592 |
| TERPENOID_BACKBONE_BIOSYNTHESIS | 0.290327 | 0.117244 |
| GLYCEROLIPID_METABOLISM | -0.28405 | 0.125367 |
| OOCYTE_MEIOSIS | 0.27722 | 0.134692 |
| O_GLYCAN_BIOSYNTHESIS | 0.269854 | 0.145347 |
| GLIOMA | 0.26856 | 0.147283 |
| OTHER_GLYCAN_DEGRADATION | 0.25566 | 0.167683 |
| NICOTINATE_AND_NICOTINAMIDE_METABOLISM | -0.2536 | 0.171131 |
| GLYCOLYSIS_GLUCONEOGENESIS | 0.249495 | 0.178154 |
| GLYCOSPHINGOLIPID_BIOSYNTHESIS_GANGLIO_SERIES | 0.248254 | 0.180319 |
| CYTOSOLIC_DNA_SENSING_PATHWAY | 0.243004 | 0.189697 |
| UBIQUITIN_MEDIATED_PROTEOLYSIS | 0.242303 | 0.190975 |
| B_CELL_RECEPTOR_SIGNALING_PATHWAY | 0.240565 | 0.194173 |
| CIRCADIAN_RHYTHM_MAMMAL | -0.24052 | 0.194263 |
| RIBOFLAVIN_METABOLISM | -0.23873 | 0.197586 |
| ALANINE_ASPARTATE_AND_GLUTAMATE_METABOLISM | -0.23619 | 0.202396 |
| P53_SIGNALING_PATHWAY | 0.233737 | 0.20712 |
| GLYCOSYLPHOSPHATIDYLINOSITOL_GPI_ANCHOR_BIOSYNTHESIS | -0.22789 | 0.218687 |
| BIOSYNTHESIS_OF_UNSATURATED_FATTY_ACIDS | 0.227347 | 0.219794 |
| DORSO_VENTRAL_AXIS_FORMATION | -0.2233 | 0.228101 |
| CHEMOKINE_SIGNALING_PATHWAY | 0.222845 | 0.229051 |
| LYSOSOME | 0.22213 | 0.230548 |
| SYSTEMIC_LUPUS_ERYTHEMATOSUS | -0.21954 | 0.236022 |
| METABOLISM_OF_XENOBIOTICS_BY_CYTOCHROME_P450 | 0.219417 | 0.236286 |
| SNARE_INTERACTIONS_IN_VESICULAR_TRANSPORT | 0.214749 | 0.246395 |
| NEUROTROPHIN_SIGNALING_PATHWAY | 0.21271 | 0.250904 |
| NOD_LIKE_RECEPTOR_SIGNALING_PATHWAY | 0.212181 | 0.252083 |
| PHOSPHATIDYLINOSITOL_SIGNALING_SYSTEM | -0.21094 | 0.254876 |
| COLORECTAL_CANCER | 0.21041 | 0.25606 |
| PROPANOATE_METABOLISM | -0.20825 | 0.260966 |
| CELL_ADHESION_MOLECULES_CAMS | -0.20773 | 0.262156 |
| MELANOGENESIS | 0.206042 | 0.266053 |
| TYPE_II_DIABETES_MELLITUS | 0.204946 | 0.268603 |
| RNA_POLYMERASE | 0.197108 | 0.287321 |
| AXON_GUIDANCE | 0.196094 | 0.289806 |
| VIBRIO_CHOLERAE_INFECTION | 0.193903 | 0.295225 |
| TYROSINE_METABOLISM | -0.19378 | 0.295523 |
| PEROXISOME | 0.19321 | 0.296953 |
| AMYOTROPHIC_LATERAL_SCLEROSIS_ALS | 0.19012 | 0.30474 |
| RNA_DEGRADATION | -0.18633 | 0.314467 |
| GLYCOSAMINOGLYCAN_BIOSYNTHESIS_CHONDROITIN_SULFATE | -0.18273 | 0.323917 |
| PYRIMIDINE_METABOLISM | 0.182357 | 0.324896 |
| LONG_TERM_DEPRESSION | 0.180918 | 0.328725 |
| DRUG_METABOLISM_CYTOCHROME_P450 | 0.180342 | 0.330266 |
| NON_HOMOLOGOUS_END_JOINING | -0.17921 | 0.333312 |
| PANCREATIC_CANCER | 0.17344 | 0.349098 |
| CITRATE_CYCLE_TCA_CYCLE | -0.17171 | 0.35391 |
| RENAL_CELL_CARCINOMA | 0.169461 | 0.360259 |
| HISTIDINE_METABOLISM | -0.16659 | 0.368452 |
| ABC_TRANSPORTERS | 0.16199 | 0.381811 |
| PROTEIN_EXPORT | 0.159835 | 0.388172 |
| JAK_STAT_SIGNALING_PATHWAY | -0.15978 | 0.388323 |
| PROSTATE_CANCER | 0.157713 | 0.394499 |
| TOLL_LIKE_RECEPTOR_SIGNALING_PATHWAY | 0.15617 | 0.399141 |
| HOMOLOGOUS_RECOMBINATION | 0.151503 | 0.413373 |
| INTESTINAL_IMMUNE_NETWORK_FOR_IGA_PRODUCTION | -0.15029 | 0.417132 |
| ADHERENS_JUNCTION | -0.14839 | 0.423034 |
| LONG_TERM_POTENTIATION | 0.146782 | 0.428069 |
| TGF_BETA_SIGNALING_PATHWAY | -0.14547 | 0.432201 |
| VASCULAR_SMOOTH_MUSCLE_CONTRACTION | 0.144391 | 0.435629 |
| SPLICEOSOME | -0.14314 | 0.439617 |
| GLUTATHIONE_METABOLISM | -0.13748 | 0.457898 |
| PROGESTERONE_MEDIATED_OOCYTE_MATURATION | 0.134464 | 0.467826 |
| GALACTOSE_METABOLISM | -0.13405 | 0.469189 |
| TASTE_TRANSDUCTION | -0.13391 | 0.46965 |
| GLYCOSAMINOGLYCAN_DEGRADATION | -0.13294 | 0.472877 |
| MAPK_SIGNALING_PATHWAY | 0.13152 | 0.477624 |
| BASAL_TRANSCRIPTION_FACTORS | -0.12868 | 0.487188 |
| VEGF_SIGNALING_PATHWAY | 0.127988 | 0.489525 |
| HYPERTROPHIC_CARDIOMYOPATHY_HCM | 0.127039 | 0.49275 |
| ARACHIDONIC_ACID_METABOLISM | 0.126659 | 0.494042 |
| ACUTE_MYELOID_LEUKEMIA | -0.12617 | 0.495722 |
| ECM_RECEPTOR_INTERACTION | 0.123671 | 0.504283 |
| NATURAL_KILLER_CELL_MEDIATED_CYTOTOXICITY | 0.123618 | 0.504468 |
| STARCH_AND_SUCROSE_METABOLISM | -0.12061 | 0.514892 |
| TRYPTOPHAN_METABOLISM | 0.114388 | 0.536804 |
| GLYCEROPHOSPHOLIPID_METABOLISM | -0.11338 | 0.540381 |
| PYRUVATE_METABOLISM | 0.113031 | 0.541644 |
| EPITHELIAL_CELL_SIGNALING_IN_HELICOBACTER_PYLORI_INFECTION | 0.110697 | 0.550021 |
| GNRH_SIGNALING_PATHWAY | -0.10858 | 0.557659 |
| FC_GAMMA_R_MEDIATED_PHAGOCYTOSIS | -0.10737 | 0.562073 |
| PARKINSONS_DISEASE | -0.10503 | 0.570619 |
| AMINOACYL_TRNA_BIOSYNTHESIS | -0.10342 | 0.576527 |
| ENDOMETRIAL_CANCER | 0.102249 | 0.580863 |
| N_GLYCAN_BIOSYNTHESIS | -0.09771 | 0.597762 |
| WNT_SIGNALING_PATHWAY | 0.096821 | 0.601098 |
| FOCAL_ADHESION | 0.095872 | 0.604669 |
| PRION_DISEASES | 0.09426 | 0.610756 |
| LEISHMANIA_INFECTION | 0.093804 | 0.612484 |
| DILATED_CARDIOMYOPATHY | 0.092738 | 0.616528 |
| PURINE_METABOLISM | 0.091294 | 0.622027 |
| VIRAL_MYOCARDITIS | -0.09104 | 0.622998 |
| LYSINE_DEGRADATION | -0.09033 | 0.625699 |
| PENTOSE_PHOSPHATE_PATHWAY | 0.090176 | 0.626298 |
| T_CELL_RECEPTOR_SIGNALING_PATHWAY | 0.088413 | 0.63306 |
| ENDOCYTOSIS | 0.082965 | 0.654145 |
| ALZHEIMERS_DISEASE | -0.07977 | 0.666641 |
| RIG_I_LIKE_RECEPTOR_SIGNALING_PATHWAY | 0.078014 | 0.67355 |
| ETHER_LIPID_METABOLISM | -0.07662 | 0.679062 |
| GLYOXYLATE_AND_DICARBOXYLATE_METABOLISM | -0.06543 | 0.72383 |
| ERBB_SIGNALING_PATHWAY | 0.065311 | 0.724319 |
| MTOR_SIGNALING_PATHWAY | 0.062839 | 0.734353 |
| BETA_ALANINE_METABOLISM | -0.05982 | 0.746675 |
| THYROID_CANCER | 0.059576 | 0.747663 |
| FC_EPSILON_RI_SIGNALING_PATHWAY | 0.056103 | 0.761911 |
| RETINOL_METABOLISM | 0.055327 | 0.765107 |
| ONE_CARBON_POOL_BY_FOLATE | -0.05282 | 0.775464 |
| ARRHYTHMOGENIC_RIGHT_VENTRICULAR_CARDIOMYOPATHY_ARVC | 0.050237 | 0.786167 |
| LEUKOCYTE_TRANSENDOTHELIAL_MIGRATION | 0.046986 | 0.799699 |
| STEROID_BIOSYNTHESIS | 0.045525 | 0.805798 |
| PPAR_SIGNALING_PATHWAY | 0.044591 | 0.809707 |
| DRUG_METABOLISM_OTHER_ENZYMES | -0.04455 | 0.809888 |
| COMPLEMENT_AND_COAGULATION_CASCADES | -0.04111 | 0.824301 |
| CHRONIC_MYELOID_LEUKEMIA | 0.038353 | 0.835916 |
| REGULATION_OF_ACTIN_CYTOSKELETON | 0.035442 | 0.848212 |
| ARGININE_AND_PROLINE_METABOLISM | 0.035193 | 0.849267 |
| ANTIGEN_PROCESSING_AND_PRESENTATION | 0.0306 | 0.868746 |
| SPHINGOLIPID_METABOLISM | -0.0304 | 0.86961 |
| APOPTOSIS | 0.029888 | 0.871774 |
| PATHWAYS_IN_CANCER | 0.028583 | 0.877327 |
| CALCIUM_SIGNALING_PATHWAY | 0.025734 | 0.889469 |
| INSULIN_SIGNALING_PATHWAY | 0.024779 | 0.893547 |
| FATTY_ACID_METABOLISM | -0.02179 | 0.906319 |
| SMALL_CELL_LUNG_CANCER | 0.020075 | 0.913667 |
| PATHOGENIC_ESCHERICHIA_COLI_INFECTION | -0.0193 | 0.916998 |
| BASAL_CELL_CARCINOMA | -0.01572 | 0.932346 |
| CARDIAC_MUSCLE_CONTRACTION | -0.01429 | 0.938466 |
| HUNTINGTONS_DISEASE | 0.012491 | 0.946217 |
| OXIDATIVE_PHOSPHORYLATION | -0.00887 | 0.96181 |
| CYTOKINE_CYTOKINE_RECEPTOR_INTERACTION | -0.007 | 0.969861 |
| TIGHT_JUNCTION | -0.00661 | 0.971527 |
| ADIPOCYTOKINE_SIGNALING_PATHWAY | 0.002629 | 0.988674 |
| NEUROACTIVE_LIGAND_RECEPTOR_INTERACTION | -0.00094 | 0.995934 |
