# Supplementary figures and images for: Histone chaperone ASF1A accelerates chronic myeloid leukemia blast crisis by activating Notch signaling
Source: Cell Death Dis. 2022 Oct 3;13(10):842. doi: 10.1038/s41419-022-05234-5 (PMC9527247; doi:10.1038/s41419-022-05234-5)

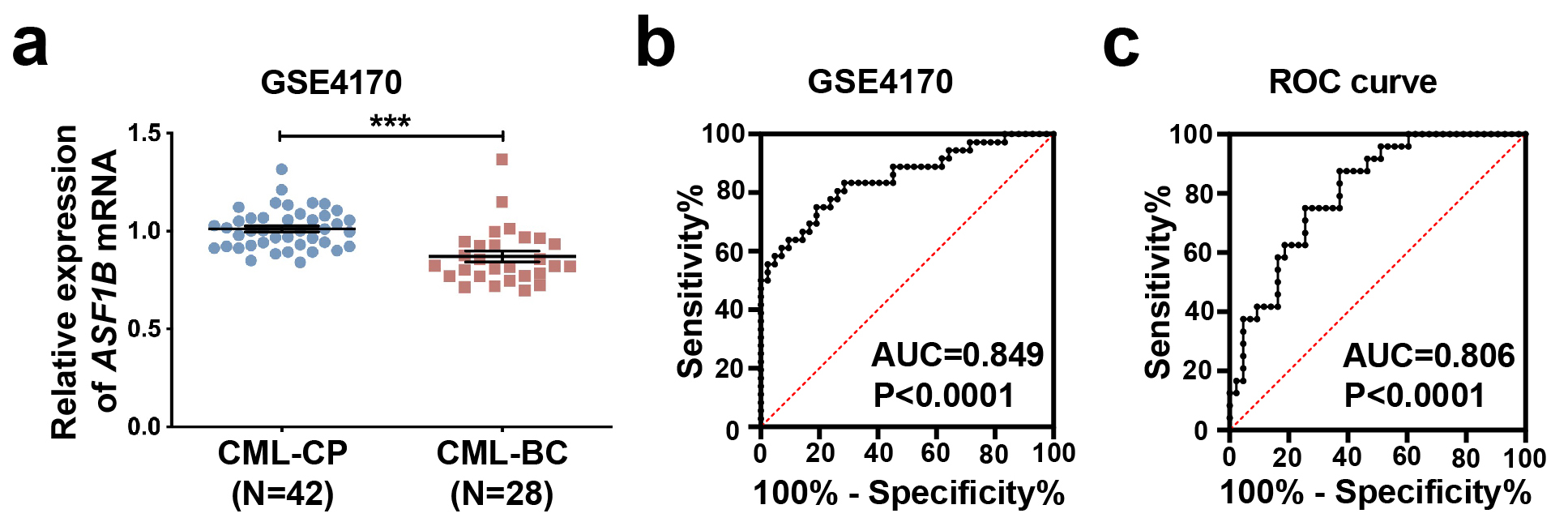

Supplement: Supplementary file 2 — Supplemental Figure.S1 [file 41419_2022_5234_MOESM2_ESM.jpg]

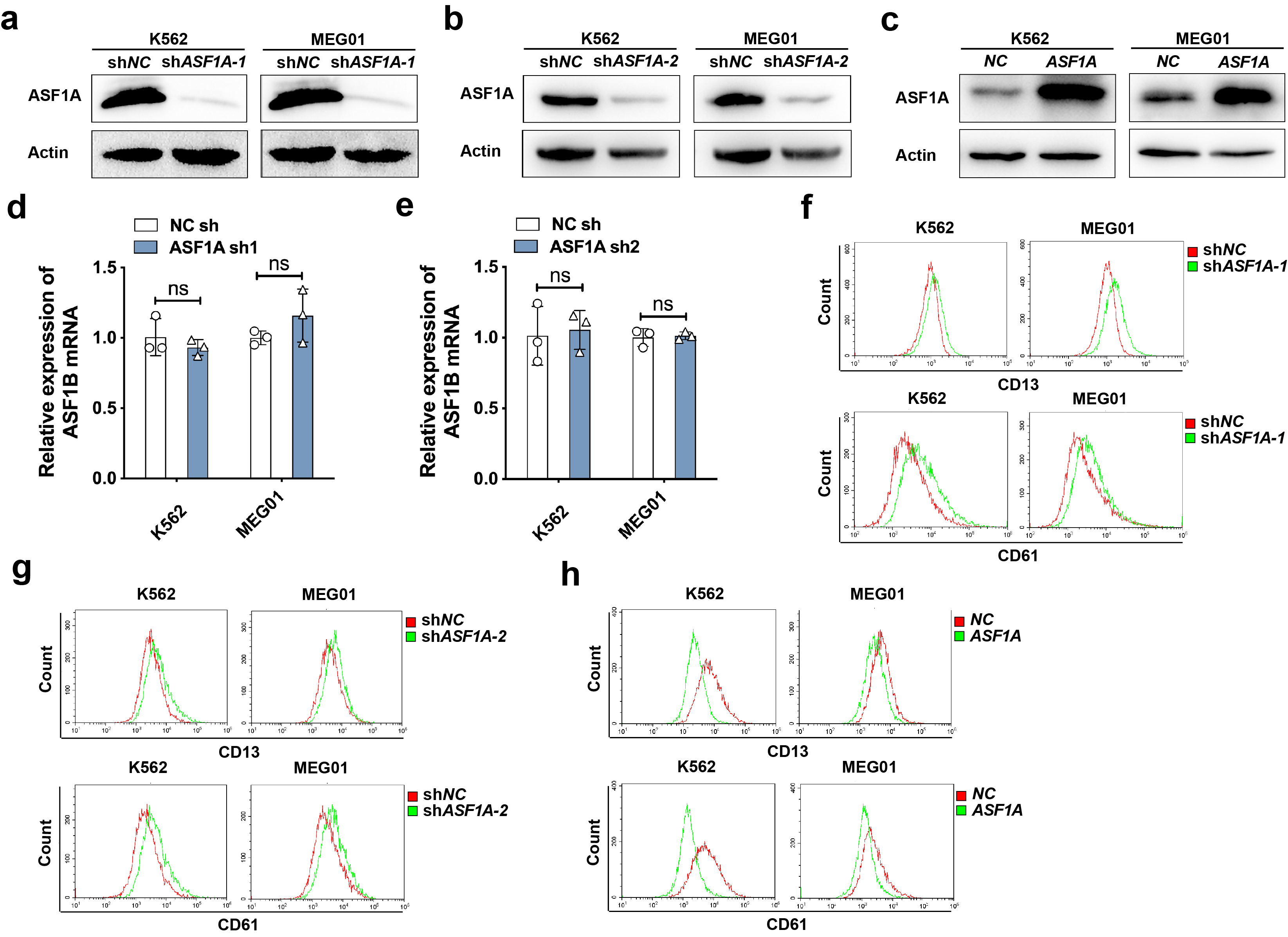

Supplement: Supplementary file 3 — Supplemental Figure.S2 [file 41419_2022_5234_MOESM3_ESM.jpg]

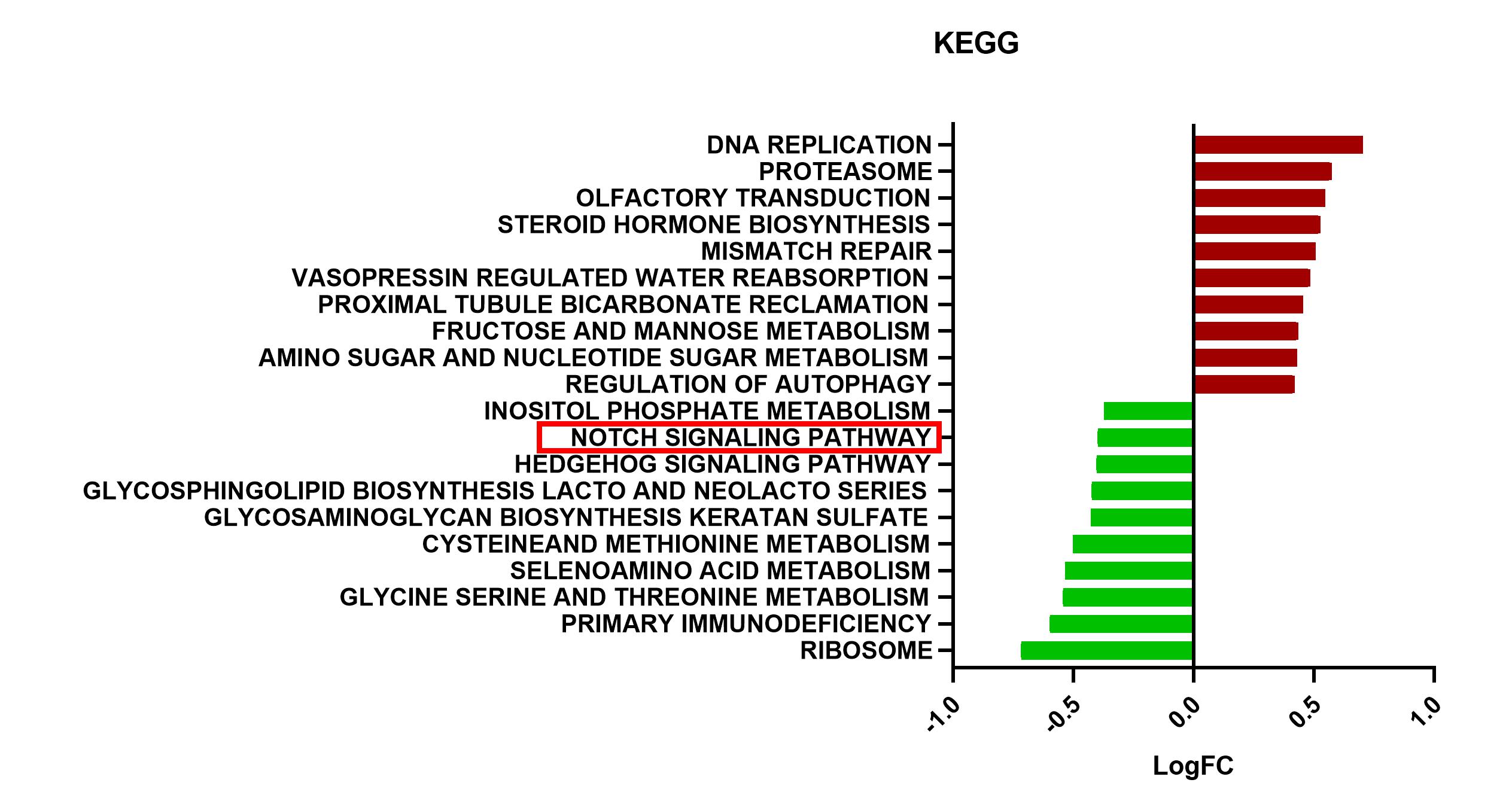

Supplement: Supplementary file 4 — Supplemental Figure.S3 [file 41419_2022_5234_MOESM4_ESM.jpg]

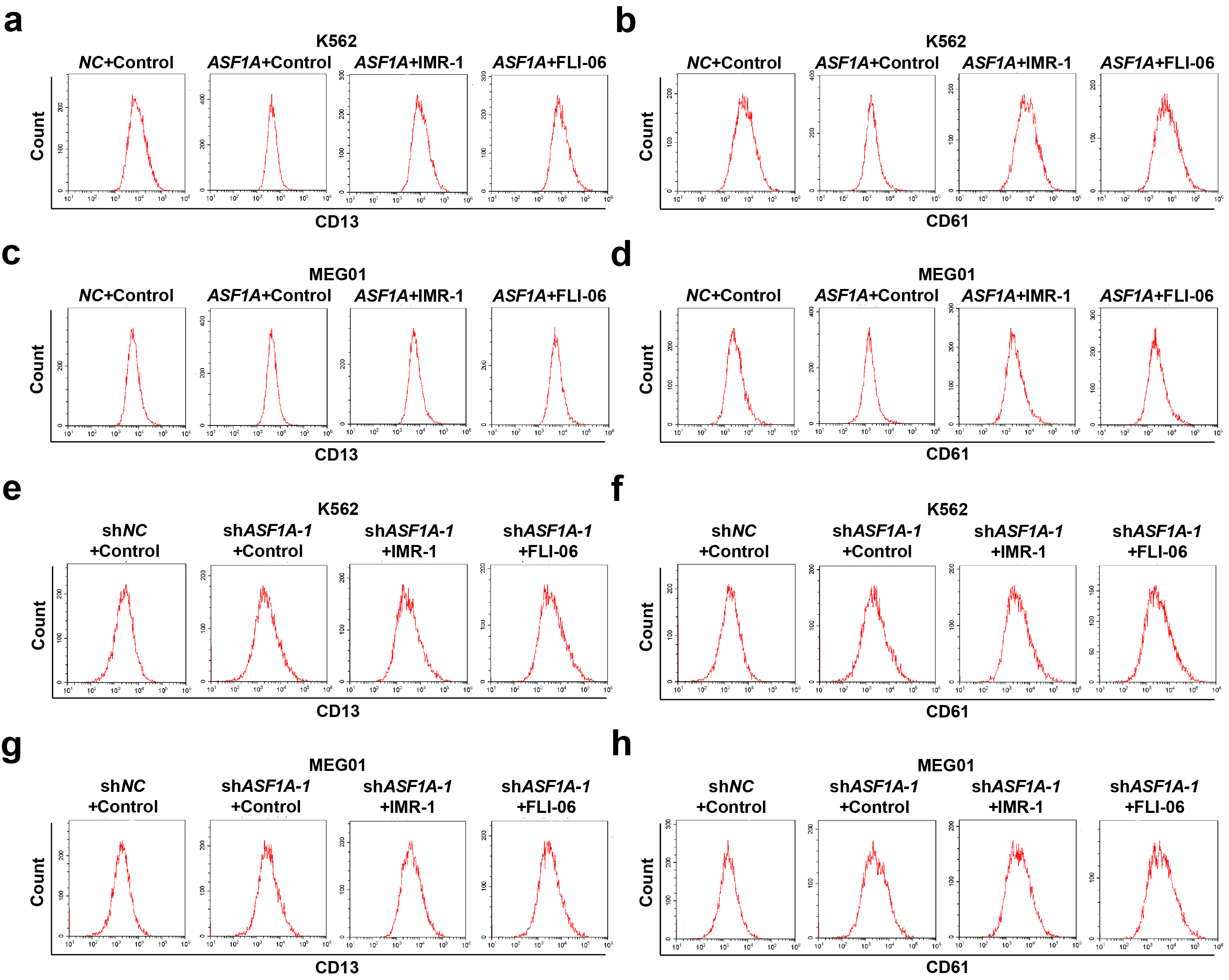

Supplement: Supplementary file 5 — Supplemental Figure.S4 [file 41419_2022_5234_MOESM5_ESM.jpg]

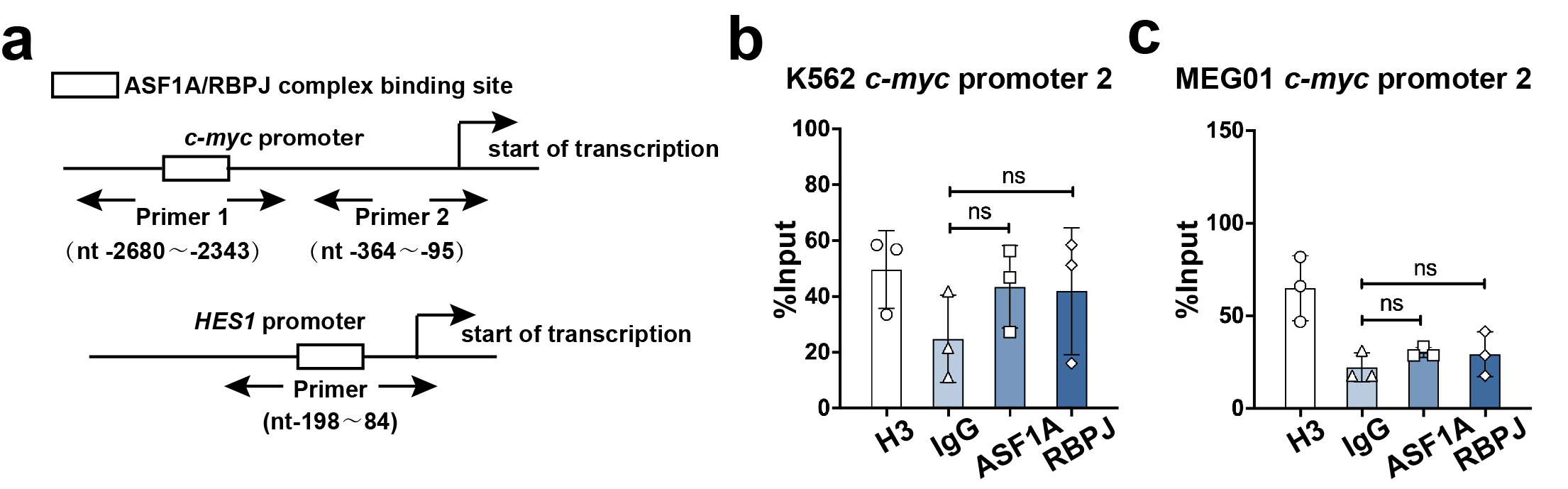

Supplement: Supplementary file 6 — Supplemental Figure.S5 [file 41419_2022_5234_MOESM6_ESM.jpg]

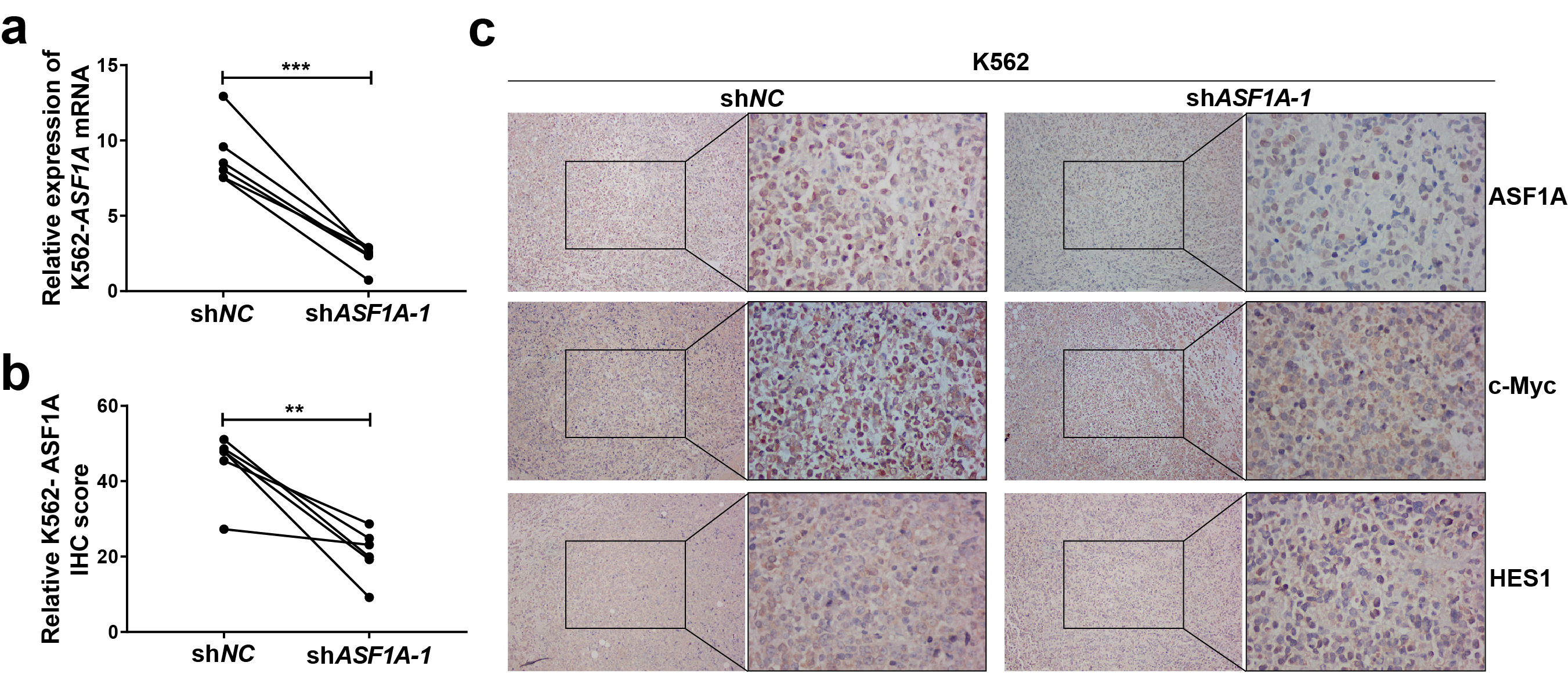

Supplement: Supplementary file 7 — Supplemental Figure.S6 [file 41419_2022_5234_MOESM7_ESM.jpg]

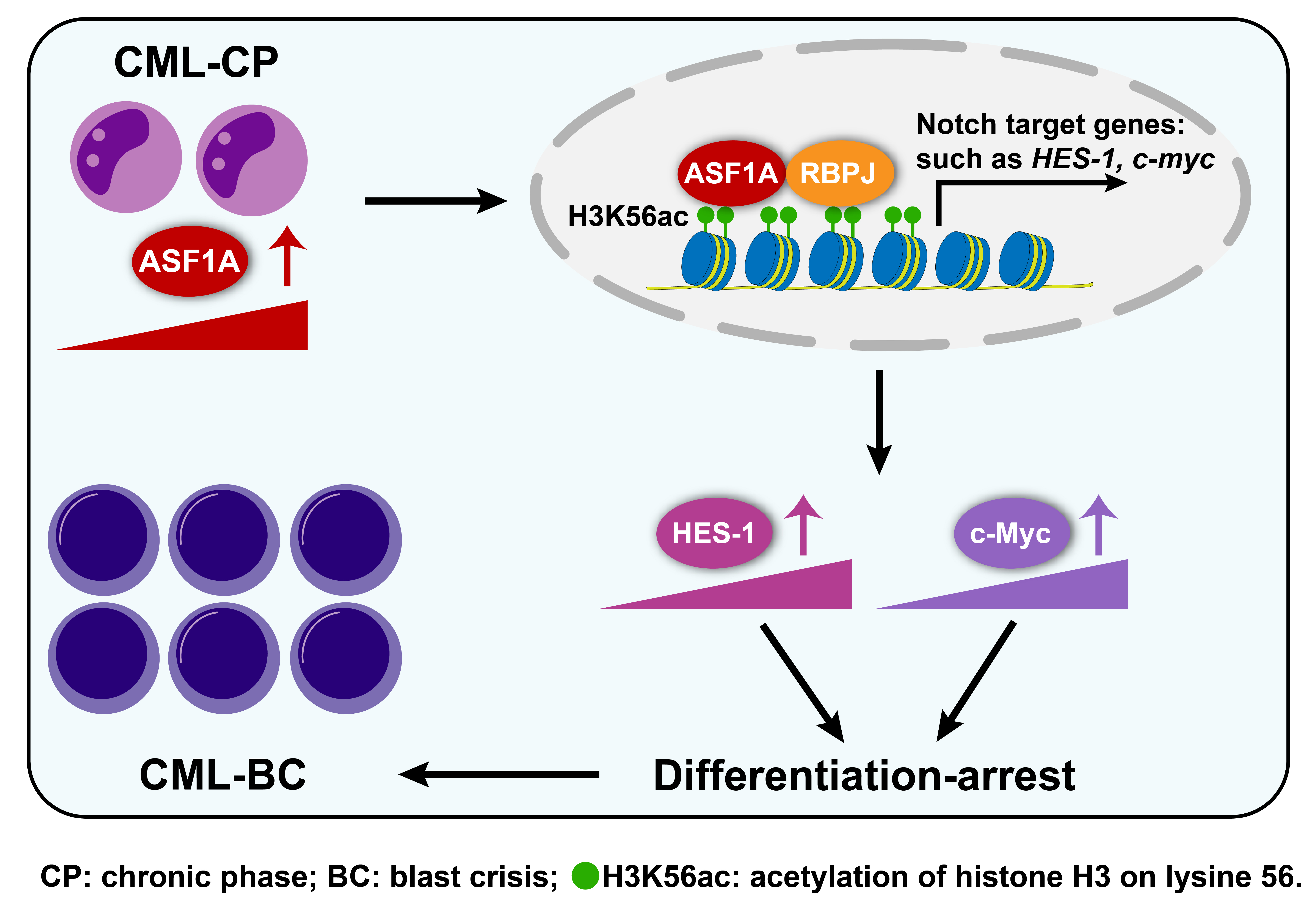

Supplement: Supplementary file 8 — Supplemental Figure.S7 [file 41419_2022_5234_MOESM8_ESM.jpg]
